# DDX3 Represses Stemness by Epigenetically Modulating Tumor-suppressive miRNAs in Hepatocellular Carcinoma

**DOI:** 10.1038/srep28637

**Published:** 2016-06-27

**Authors:** Hao-Kang Li, Ru-Tsun Mai, Hsien-Da Huang, Chih-Hung Chou, Yi-An Chang, Yao-Wen Chang, Li-Ru You, Chun-Ming Chen, Yan-Hwa Wu Lee

**Affiliations:** 1 Institute of Biochemistry and Molecular Biology, School of Life Sciences, National Yang-Ming University, Taipei, Taiwan; 2 Department of Biological Science and Technology, College of Biological Science and Technology, National Chiao Tung University, Hsinchu, Taiwan; 3 Institute of Bioinformatics and Systems Biology, College of Biological Science and Technology, National Chiao Tung University, Hsinchu, Taiwan; 4 Department of Medical Research, Mackay Memorial Hospital, Hsinchu, Taiwan; 5 Department of Life Sciences and Institute of Genome Sciences, School of Life Sciences, National Yang-Ming University, Taipei, Taiwan

## Abstract

Studies indicate that the presence of cancer stem cells (CSCs) is responsible for poor prognosis of hepatocellular carcinoma (HCC) patients. In this study, the functional role of DDX3 in regulation of hepatic CSCs was investigated. Our results demonstrated that reduced DDX3 expression was not only inversely associated with tumor grade, but also predicted poor prognosis of HCC patients. Knockdown of DDX3 in HCC cell line HepG2 induced stemness gene signature followed by occurrence of self-renewal, chemoreisistance, EMT, migration as well as CSC expansion, and most importantly, DDX3 knockdown promotes tumorigenesis. Moreover, we found positive correlations between DDX3 level and expressions of tumor-suppressive miR-200b, miR-200c, miR-122 and miR-145, but not miR-10b and miR-519a, implying their involvement in DDX3 knockdown-induced CSC phenotypes. In addition, DDX3 reduction promoted up-regulation of DNA methyltransferase 3A (DNMT3A), while neither DNMT3B nor DNMT1 expression was affected. Enriched DNMT3A binding along with hypermethylation on promoters of these tumor-suppressive miRNAs reflected their transcriptional repressions in DDX3-knockdown cells. Furthermore, individual restoration of these tumor-suppressive miRNAs represses DDX3 knockdown-induced CSC phenotypes. In conclusion, our study suggested that DDX3 prevents generation of CSCs through epigenetically regulating a subset of tumor-suppressive miRNAs expressions, which strengthens tumor suppressor role of DDX3 in HCC.

Over the past few decades, accumulating evidence supports that a single cell derived from different cancers gives rise to hierarchic organization within a tumor, which has emerged as cancer stem cell (CSC) model^1^. Like normal stem cells, the stem-like cells at the apex of CSC model self-renew and differentiate, which contribute to the heterogeneity observed in the clonally derived tumors. Moreover, these stem-like cells are highly chemoresistant and metastatic^2^. Thus, the presence of CSCs in tumors predicts poor prognosis of cancer patients, and therapeutic strategies targeting CSCs provide efficacy to eradicate cancers^3^.

Recent studies show that certain microRNAs (miRNAs) exhibit promising therapeutic potential by suppressing both cancer cells and CSCs^4^. miRNAs are a group of ~22-nucleotide non-coding single-stranded RNAs involved in a myriad physiological functions, including cell proliferation, survival, metabolism, differentiation and invasion^5^. In previous studies, miRNAs have been linked to regulation of self-renewal and differentiation of embryonic stem cells (ESCs). More recently, it is also shown that deregulation of miRNAs results in gains of CSC properties in several types of cancers^6^. For example, miRNA profiling indicates that marked down-regulation of tumor-suppressive miR-200b, miR-200c and miR-145 causes overexpression of pluripotency-associated factors, such as Nanog, Oct4, c-Myc, Sox2 and KLF4, and components of polycomb repressive complex like Bmi1^7^, thereby conferring the abilities of self-renewal, metastasis and chemoresistance on CSCs^8^,^9^,^10^. In hepatocellular carcinoma (HCC), loss of liver abundant miR-122 suggests its essential role to maintain hepatic phenotypes and prevents tumor progression from expansion of CSC populations^11^,^12^,^13^. These CSCs in HCC are defined by functional properties and a panel of surface antigens, such as CD133, CD13, epithelial cell adhesion molecule (EpCAM) and CD90^14^. Furthermore, acquisition of CSC phenotypes, including epithelial-mesenchymal transition (EMT), invasion and chemoresistance, are also linked to the reduction of miR-200b, miR-200c and miR-145 in HCC^15^,^16^,^17^. In this regard, the deregulation of miRNAs leading to the generation of CSCs in HCC may explain the high recurrence rate of this deadly disease^18^.

miRNA biogenesis includes transcription, Drosha complex-mediated processing of primary transcript (pri-miRNA) to precursor miRNA (pre-miRNA), exportin 5-facilitated nuclear export of pre-miRNAs, and Dicer-regulated processing of pre-miRNA to mature miRNA^19^. In addition to chromosomal abnormalities and DNA mutations, epigenetic deregulation of miRNA gene promoters or aberrant expression of the genes involved in the biogenesis pathways have been described in numerous types of cancer^5^,^19^. Several DNA methyltransferases (DNMTs), including DNMT3A, DNMT3B and DNMT1, play pivotal roles in establishing and maintaining the methylation patterns of genomic regions^20^. The DNA hypermethylation at CpG island in promoter regions of tumor-suppressive miRNAs are crucial for silencing their transcriptions^21^. For example, hypermethylation of miR-200b and miR-200c promoter regions repress their transcriptions, and are associated with occurrence of EMT and acquisition of stem cell-like properties during cell transformation^22^. Characterization in metastatic cells indicates that reversion of miR-145 promoter hypermethylation up-regulates its expression along with reduced expression of Oct4 and c-Myc levels^23^. During differentiation of human ESCs into hepatocytes, demethylation of miR-122 promoter initiates its transcription and subsequently inhibits self-renewal capacity^24^. Altogether, these regulations reinforce the link between deregulation of miRNAs and induction of stemness.

DEAD-box RNA helicases have multiple functions in RNA metabolism such as regulation of transcription, splicing, mRNA export, translation, RNA decay, ribosome biogenesis and miRNA regulation^19^,^25^,^26^. DDX3, a member of the DEAD-box RNA helicase family, is ubiquitously expressed in a broad range of tissues to control pleiotropic physiological events^27^,^28^. Previous studies show that DDX3 plays an oncogenic role in tumorigenesis by promoting transformation, invasion and cell growth^29^,^30^,^31^. Recent studies further demonstrate DDX3 overexpression in murine melanoma CD133^+^ CSC population^32^, and this overexpression induces CSC properties in human lung cancer cells^33^. Other than oncogenic functions, DDX3 also acts as a tumor suppressor. Our studies indicate that the reduction of DDX3 in HCC is correlated with male gender and HBV infection^34^, and its overexpression inhibits colony expansion through activation of p21^waf1/cip1^ transcription^35^. p21^waf1/cip1^ activation by DDX3 also occurs in lung cancer^36^, and loss of DDX3 confers cells with the ability of anchorage-independent growth and invasion^37^. Despite aberrant expression of DDX3 in different cancers, its interaction with cellular factors exerts regulatory function on tumorigenic process as well^27^. Moreover, association of DDX3 with components of pri-miRNA processing complex suggests its involvement in miRNA biogenesis^19^,^38^. However, whether DDX3 participates in the regulation of tumorigenesis through modulation of miRNA expressions is still unclear.

In this study, we provide evidences that DDX3 acts as a tumor suppressor to inhibit CSC phenotypes in HCC. Our results indicate that reduced DDX3 correlates with poor survival in HCC patients, and promotes stemness in HCC cell line HepG2. Moreover, down-regulation of DDX3 induces stem cell-like properties, including self-renewal, chemoresistance, EMT, motility and CSC expansion, which gave rise to tumor initiation. Further characterization reveals that knockdown of DDX3 is associated with overexpression of DNMT3A and down-regulation of tumor-suppressive miR-200b, miR-200c, miR-122 and miR-145 through enriched DNMT3A binding and enhanced cytosine methylation as well as H3K27me3 elevation on promoter regions of these tumor-suppressive miRNAs. In addition, these miRNAs individually mediated suppression of CSC phenotypes in DDX3-knockdown cells. These findings suggest that DDX3 epigenetically regulates expression of a subset of tumor-suppressive miRNAs to inhibit expansion of hepatic CSCs. Taken together, our findings strengthens the tumor suppressor role of DDX3 in HCC, which would be helpful in the development of new therapeutic strategy for eradication of hepatic CSCs.

## Results

### Reduced DDX3 expression is associated with higher tumor grade and lower survival probability in HCC patients

**Figure 1:**
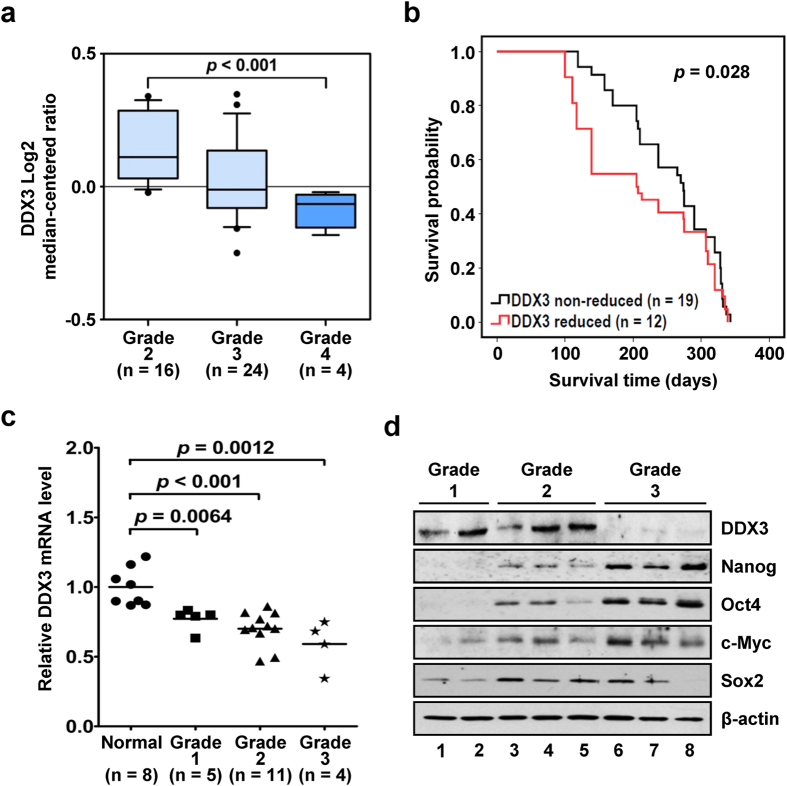
Decreased expression of DDX3 is associated with poor differentiation of tissue in HCC patients. (**a**) High HCC grade showed significant reduction of DDX3. Expression level of DDX3 in HCC tissues of total 44 samples extracted from the *Ye* dataset in the cancer clinical microarray database *Oncomine* were grouped according to histological grade (Grade 2, 3 and 4), and number of samples in each grade is shown. DDX3 expression is presented as Log2 median-centered ratio. The statistical result of DDX3 level with different HCC grade was analyzed by outlier analysis. (**b**) Poor survival probability was observed in HCC patients with reduced DDX3 expression. The patients in the *Ye* dataset were grouped by non-reduced and reduced expression of DDX3, and their survival probability was assessed by Kaplan-Meier estimator. *p* < 0.05 represents statistical significance. (**c**) DDX3 expression decreases with the progression of HCC. DDX3 mRNA level in normal as well as HCC tissues of Grade 1, 2 and 3 acquired from OriGene TissueScan Liver Cancer cDNA Array was evaluated by qRT-PCR. Level of DDX3 mRNA in HCC samples was transformed into fold change relative to that of normal samples. Statistical analyses were carried out by *t* test. *p* < 0.05 represents statistical significance. (**d**) High HCC grade was associated with down-regulation of DDX3 and up-regulation of pluripotency factors. Lysates (100 μg each) of representative HCC tissues acquired from TLCN were analyzed by immunoblotting using anti-DDX3, Nanog, Oct4, c-Myc, Sox2 and β-actin antibodies.

To investigate possible link between DDX3 expression and liver cancer progression, an *in silico* analysis of DDX3 transcript level grouped according to tumor grade was performed with a published HCC clinical microarray dataset in the public database *Oncomine*^39^, and 44 tissues derived from 31 patients were included in the present study. In Fig. 1a, DDX3 expression was decreased in Grade 3 and 4 HCC tissues as compared with that of Grade 2, and a significant reduction between Grade 2 and Grade 4 was observed (*p* < 0.001). Moreover, reduced DDX3 expression was associated with a poor survival probability in the same cohort of patients (Fig. 1b, *p* = 0.028). The median survival time was 225 days for patients with reduced DDX3 expression (n = 12) and 290 days for patients with non-reduced DDX3 expression (n = 19). This correlation of lower DDX3 expression with higher HCC grade was verified in a commercial liver cancer cDNA array and HCC tissues acquired from Taiwan Liver Cancer Network (TLCN) (see Materials and methods). As shown in Fig. 1c, a gradual and significant decrease in DDX3 mRNA level was noted as HCC tumor grade increased. Also, protein level of DDX3 was significant reduced in Grade 3 HCC tissues compared to that of Grade 1 and 2 HCC tissues (Fig. 1d). In addition, as compared with Grade 1 HCC tissues, higher protein expressions of several stem cell transcription factors, including Nanog, Oct4, c-Myc and Sox2, in Grade 2 and 3 HCC tissues were observed, which reflected the relatively poor differentiation status of these HCC tissues (Fig. 1d). Therefore, these results indicated that the reduction in DDX3 expression was associated with poor differentiation status of HCC tissues and lower survival probability of HCC patients.

### DDX3 represses expression of stemness gene signature

**Figure 2:**
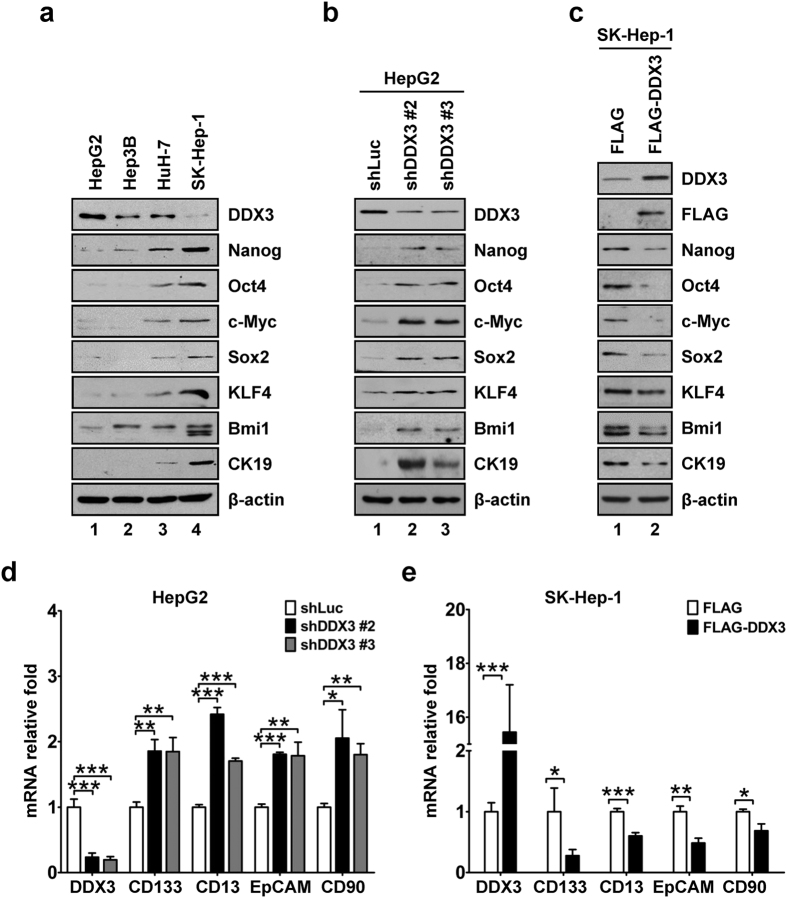
DDX3 suppresses stemness gene signature. (**a**) Lower expression of DDX3 along with overexpression of stemness markers was observed in the poorly differentiated cell line. Cell lysates (50 μg) of HepG2, Hep3B, HuH-7 and SK-Hep-1 cells were analyzed by western blotting with antibodies against DDX3, Nanog, Oct4, c-Myc, Sox2, KLF4, Bmi1, CK19 and β-actin. (**b**) Knockdown of DDX3 led to up-regulation of stemness markers. Cell lysates (50 μg) of stable shLuc, shDDX3 #2 and shDDX3 #3 HepG2 cells were analyzed by western blotting with antibodies described in (**a**). (**c**) DDX3 overexpression suppressed stemness markers. Plasmid pcDNA3-SRα/FLAG or pcDNA3-SRα/FLAG-DDX3 was transfected into SK-Hep-1 cells. At 48 h post transfection, cell lysates were prepared and subjected to immunoblotting with antibodies described in (**a**) and anti-FLAG antibody. β-actin was used as internal control in (**a**–**c**). (**d**) DDX3 knockdown was correlated with up-regulation of hepatic CSC surface markers. mRNA expressions of DDX3, CD133, CD13, EpCAM, CD90 and GAPDH in shLuc, shDDX3 #2 and shDDX3 #3 cells were detected by qRT-PCR. GAPDH was used as internal control. Fold change of each mRNA transcript in shDDX3 #2 and shDDX3 #3 cells was relative to that of shLuc cells. (**e**) DDX3 overexpression resulted in suppression of hepatic CSC surface markers. SK-Hep-1 cells were transfected with plasmid pcDNA3-SRα/FLAG or pcDNA3-SRα/FLAG-DDX3 as described in (**c**). At 48 h post transfection, total RNA was extracted and subjected to qRT-PCR analysis. GAPDH was used as internal control. Fold change of each mRNA transcript in FLAG-DDX3-expressing cells was relative to that of vector control cells. All experiments were performed at least three times, and the error bar indicates ± 1 s.d. of the mean. Statistical analyses were carried out using *t* test (*p < 0.05; **p < 0.01; ***p < 0.001).

As lower DDX3 level was correlated with poor differentiation status in clinical HCC samples, we validated the association of DDX3 expression with differentiation and stemness gene signature in three well differentiated (HepG2, Hep3B and HuH-7) and one poorly differentiated (SK-Hep-1) liver cancer cell lines. Among these cell lines, well differentiated HepG2 cells showed higher DDX3 expression along with lower protein levels of stemness gene signature, including Nanog, Oct4, c-Myc, Sox2, KLF4, Bmi1 and CK19, while lower DDX3 level and higher expressions of stemness gene signature were observed in poorly differentiated SK-Hep-1 cells (Fig. 2a). As compared with HepG2 cells, both Hep3B and HuH-7 cells showed slight reduction in DDX3 accompanied with modest increase in Nanog and Bmi1 (Fig. 2a). Moreover, enhanced expression of the rest of the stemness gene signature was observed in HuH-7 cells as compared with that of HepG2 cells, whereas weak or undetectable level was shown in Hep3B cells (Fig. 2a). To further delineate the association between DDX3 and stemness genes expression, stable DDX3-knockdown HepG2 cells or transient DDX3-overexpressing SK-Hep-1 cells were employed. In comparison with control (shLuc) cells, stable DDX3-knockdown (shDDX3 #2 and shDDX3 #3) HepG2 cells exhibited up-regulation of Nanog, Oct4, c-Myc, Sox2, KLF4, Bmi1 and CK19 (Fig. 2b). Moreover, when FLAG-DDX3-expressing construct was transfected into SK-Hep-1 cells, protein levels of these stemness genes were down-regulated as compared with that of vector control cells (Fig. 2c). Thus, these observations suggested an inverse association of DDX3 with stemness gene signature. In addition, the role of DDX3 in the expression of hepatic CSC surface markers was also evaluated. As shown in Fig. 2d,e, mRNA expressions of CD133, CD13, EpCAM and CD90 were enhanced in stable DDX3-knockdown (shDDX3 #2 and shDDX3 #3) HepG2 cells and suppressed in FLAG-DDX3-overexpressing SK-Hep-1 cells as compared with those of their corresponding control cells. Taken together, these findings indicated that DDX3 exerted a suppressive function on the expression of stemness genes and hepatic CSC surface markers.

### DDX3 knockdown promotes stem cell-like properties as well as expansion of hepatic CSC populations

**Figure 3:**
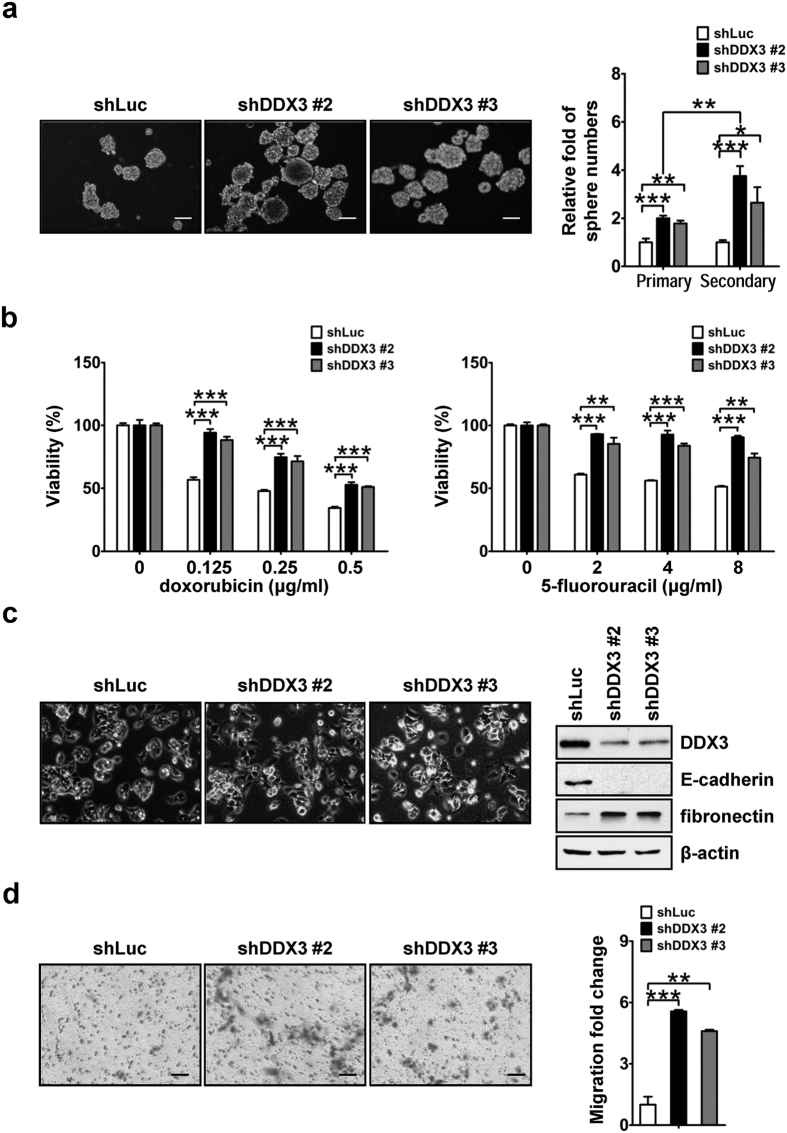
Down-regulation of DDX3 enhances cell capabilities of self-renewal, chemoresistance, EMT and migration. (**a**) DDX3 knockdown promoted self-renewal capability. Sphere formation assays of shLuc, shDDX3 #2 and shDDX3 #3 cells were performed and images of formed sphere were captured as described in the Materials and methods. Scale bar represents 100 μm. The primary spheres of these cells were further dissociated into single cells by trypsinization and subjected to another round of sphere formation assay. The numbers of primary and secondary spheres in shDDX3 #2 and shDDX3 #3 cells were transformed into fold change relative to that of shLuc cells. (**b**) Cells with decreasing DDX3 expression were more viable upon conventional anti-cancer drugs treatment. shLuc, shDDX3 #2 and shDDX3 #3 cells were treated with different concentrations of doxorubicin (0, 0.125, 0.25 and 0.5 μg/ml; left panel) or 5-fluorouracil (0, 2, 4 and 8 μg/ml; right panel), and cell viability was determined by MTT assay. Formazan absorbance at 550 nm of untreated cells was arbitrarily set as 100% viable. Viability of shLuc, shDDX3 #2 and shDDX3 #3 cells at each drug concentration was relative to that of corresponding untreated cells. (**c**) DDX3 knockdown promoted EMT. Microscopy of shLuc, shDDX3 #2 and shDDX3 #3 cells are shown. Scale bar is equal to 100 μm. Cell lysates (50 μg) of shLuc, shDDX3 #2 and shDDX3 #3 cells were analyzed by western blotting with antibodies against DDX3, E-cadherin, fibronectin and β-actin. (**d**) Down-regulation of DDX3 promoted cell mobility. Migration assays of shLuc, shDDX3 #2 and shDDX3 #3 cells were carried out and images of Giemsa-stained migrated cells were captured. Scale bar represents 100 μm. The number of migrated cells in shDDX3 #2 and shDDX3 #3 cells were relative to that in shLuc cells. All experiments were repeated at least three times, and the error bar indicated ± 1 s.d. of the mean. Statistical analyses were carried out using *t* test (*p < 0.05; **p < 0.01; ***p < 0.001).

As noted, overexpression of stemness genes confers cells with stem cell-like properties^14^. Self-renewal is pivotal for expanding and maintaining populations of stem cells as well as CSCs^14^. To investigate the role of DDX3 in the regulation of self-renewal, we performed serial sphere formation assay with shLuc, shDDX3 #2 and shDDX3 #3 HepG2 cells. As shown in Fig. 3a, primary sphere formation with shDDX3 #2 and shDDX3 #3 cells showed 1.8- to 2.0-fold enhancement as compared with that of shLuc cells. Cells derived from primary spheres were used to generate secondary spheres, and a greater enrichment (2.5- to 3.5-fold increase) of sphere numbers was observed in DDX3-knockdown cells compared to that of control cells. Thus, enhanced serial sphere-forming capability in DDX3-knockdown HepG2 cells suggested that DDX3 suppressed self-renewal capability in liver cancer cells.

In addition to self-renewal to maintain cell populations, chemoresistance protects CSCs from cytotoxic effect of chemotherapeutic agents and makes them hard to eradicate^14^. To test the involvement of DDX3 in regulating chemosensitivity of liver cancer cells, viability of shLuc, shDDX3 #2 and shDDX3 #3 cells in the presence of two conventional anti-cancer drugs, doxorubicin and 5-fluorouracil, were examined. It was shown that shDDX3 #2 and shDDX3 #3 cells were more viable than shLuc cells at different concentration of drugs relative to their corresponding untreated cells (Fig. 3b). This result indicated that DDX3 repressed the development of chemoresistance in liver cancer cells.

EMT not only contributes to dissemination of cells during cancer progression, but also promotes self-renewal capability and chemoresistance of CSCs^40^. As shown in Fig. 3c, we noted that shDDX3 #2 and shDDX3 #3 cells showed morphological changes including cell scattering and elongated cell shape as compared with that of shLuc cells under normal passage condition. This reflected the down-regulation of epithelial marker E-cadherin and up-regulation of mesenchymal marker fibronectin observed in DDX3-knockdown HepG2 cells (Fig. 3c). Moreover, the reduction of DDX3 also enhanced (4.6- to 5.6-fold increase) migration ability of HepG2 cells (Fig. 3d). These observations suggested that DDX3 suppressed EMT and subsequent migration in liver cancer cells.

In addition to examining the effect of DDX3 knockdown on CSC phenotypes in HepG2 cells, we also analyzed several stem cell-like properties in FLAG-DDX3-overexpressing SK-Hep-1 cells. As shown in Supplementary Figure S1, DDX3 overexpression resulted in the inhibition of self-renewal, chemoreisistance, EMT and migration in SK-Hep-1 cells. These results strengthened the conclusion that DDX3 functions as a negative regulator in the induction of CSC phenotypes.

**Figure 4:**
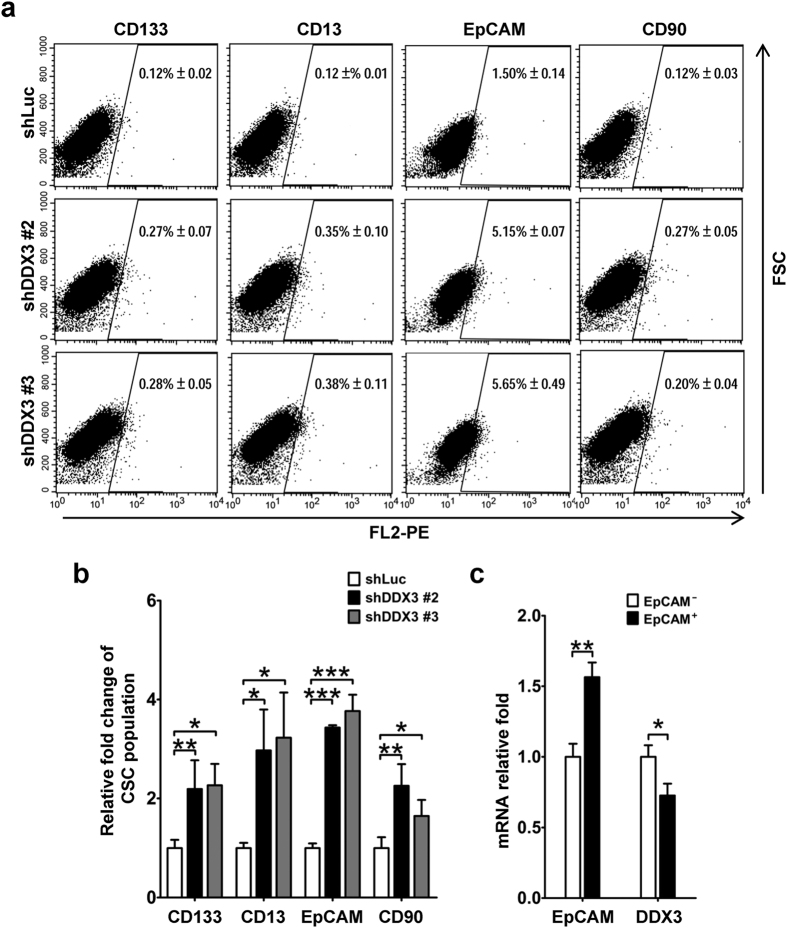
Knockdown of DDX3 enhances generation of hepatic CSC surface marker-positive populations. (**a**) DDX3 knockdown increased CD133^+^, CD13^+^, EpCAM^+^ and CD90^+^ CSC populations. PE-conjugated anti-CD133, anti-CD13, anti-EpCAM, anti-CD90 or isotype control antibodies were used to stain shLuc, shDDX3 #2 and shDDX3 #3 cells. Percentage of hepatic CSC surface marker-positive cells was determined by flow cytometry. (**b**) The percentage of CSC populations in shDDX3 #2 and shDDX3 #3 cells shown in (**a)** was transformed into fold change relative to that in shLuc cells. (**c**) DDX3 expression was reduced in EpCAM^+^ cells. Expressions of EpCAM, DDX3 and GAPDH mRNAs in EpCAM^+^ and EpCAM^−^ cells were analyzed by qRT-PCR. GAPDH was used as internal control. The amount of EpCAM or DDX3 transcript in EpCAM^+^ cells was transformed into fold change relative to that of EpCAM^−^ cells. All results were derived from at least three independent experiments, and the error bar indicated ± 1 s.d. of the mean. Statistical analyses were carried out using *t* test (*p < 0.05; **p < 0.01; ***p < 0.001).

Since the appearance of cells with stem cell-like properties implied presence of CSCs, we then examined the function of DDX3 on regulation of hepatic CSC populations. Percentage of the corresponding subpopulations in shLuc, shDDX3 #2 and shDDX3 #3 cells was analyzed by flow cytometry. As shown in Fig. 4a, expansion of CD133^+^, CD13^+^, EpCAM^+^ and CD90^+^ CSC populations was seen in DDX3-knockdown cells relative to those in shLuc cells. As compared with shLuc cells, shDDX3 #2 and shDDX3 #3 cells displayed about 1.6- to 3.7-fold increase of CD133^+^, CD13^+^, EpCAM^+^ and CD90^+^ CSC populations (Fig. 4b). Moreover, fluorescence-activated cell sorting (FACS)-isolated EpCAM^+^ population from parental HepG2 cells exhibited lower expression (28% decrease) of DDX3 mRNA than that of isolated EpCAM^−^ populations (Fig. 4c). Therefore, DDX3 expression has a negative effect on generation of hepatic CSC populations.

Taken together, all these results provided evidences that supported the inhibitory role of DDX3 in self-renewal, chemoresistance, EMT, migration and hepatic CSC population expansion.

### DDX3 knockdown initiates tumorigenesis

As down-regulation of DDX3 confers cells with stem cell-like properties and expands hepatic CSC populations, it is important to determine whether loss of DDX3 in HepG2 cells initiate tumorigenesis. To this end, we subcutaneously transplanted different numbers of shLuc, shDDX3 #2 and shDDX3 #3 cells into NOD/SCID mice (see Supplementary Figure S2). Our results demonstrated that 5 × 10^4^ and 1 × 10^5^ of DDX3-knockdown cells exhibited higher tumorigenic incidence (16.7–22.2% for shDDX3 #2 cells and 33.3–44.4% for shDDX3 #3 cells) compared to shLuc cells (0–8.3%) (Table 1). In addition, the average tumor latency of shDDX3 #2 and shDDX3 #3 cells was 2 weeks at 5 × 10^4^ or 1 × 10^5^ implanted cell density, whereas the tumor derived from 1 × 10^5^ implanted shLuc cells was observed 6 weeks post implantation. Moreover, there was no tumor developed in the groups of 1 × 10^3^ and 1 × 10^4^ implanted cells even after 25-week monitoring. These observations further supported the tumor suppressor role of DDX3 in liver cells *in vivo*.

### DDX3 expression positively correlates with transcription of tumor-suppressive miR-200b, miR-200c, miR-122 and miR-145

Emerging evidences indicate that deregulation of miRNAs leads to the appearance of cells with stem cell-like properties and plays important roles in tumorigenesis of several types of cancer, including HCC^6^,^11^,^41^,^42^. To test if miRNAs are involved in the inhibition of CSC traits induced by DDX3, a genomic profiling of miRNA expression with total RNA extracted from shLuc, shDDX3 #2 and shDDX3 #3 cells was performed (see Materials and methods). According to stem cell-like properties, we identified a list of miRNA candidates affected by DDX3 knockdown in HepG2 cells (Table 2). The experimentally validated miRNA-target interaction was manually curated from literature survey and miRTarBase^43^. Among these DDX3-responsive miRNA candidates, we found that down-regulation of miR-200b and miR-145 caused by DDX3 knockdown might reflect the association with overexpression of stemness gene signature (Fig. 2b). In addition to miR-200b and miR-145, it has been noted that suppression of miR-122 or miR-200c confers cells with CSC phenotypes in liver stem cells^13^,^17^. Therefore, these tumor-suppressive miRNAs (miR-200b, miR-200c, miR-122 and miR-145) were included in the following study.

**Figure 5:**
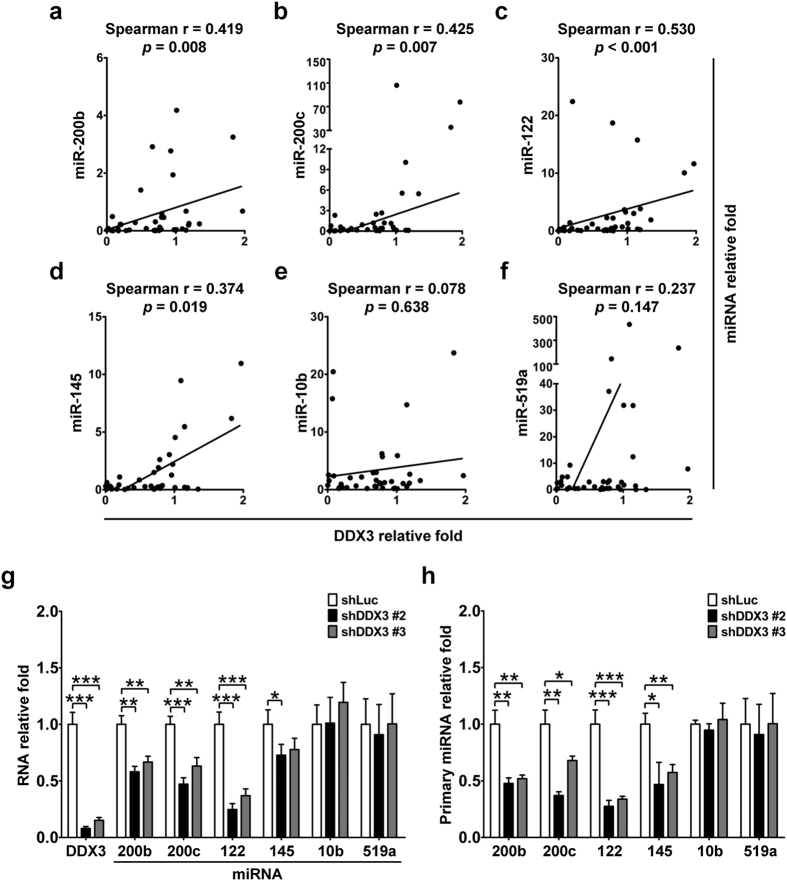
DDX3 level correlates with expression of several tumor-suppressive miRNAs. Expressions of miRNAs and U6 snRNA as well as mRNA transcripts of DDX3 and GAPDH in 39 paired tumor and non-tumor tissues of HCC patients from TLCN were analyzed by qRT-PCR. U6 snRNA and GAPDH transcripts were used as internal control for miRNA and DDX3 expression, respectively. Fold change of each transcript in tumor tissues was relative to that of corresponding non-tumor tissues. Correlation of (**a**) miR-200b, (**b**) miR-200c, (**c**) miR-122, (**d**) miR-145, (**e**) miR-10b and (**f**) miR-519a expression with DDX3 level was performed by Spearman correlation criteria. *p* < 0.05 represents statistical significance. (**g**) Knockdown of DDX3 reduced expressions of tumor-suppressive miRNAs. Total RNA extracted from shLuc, shDDX3 #2 and shDDX3 #3 cells was subjected to qRT-PCR for detection of miR-200b, miR-200c, miR-122, miR-145, miR-10b, miR-519a and U6 snRNA as well as DDX3 and GAPDH transcripts. U6 snRNA and GAPDH transcripts were served as internal control for miRNA and DDX3 expression, respectively. Fold change of each transcript in shDDX3 #2 and shDDX3 #3 cells was relative to that of shLuc cells. Experiments were performed at least three times, and the error bar indicates ± 1 s.d. of the mean. Statistical analyses were carried out using *t* test (*p < 0.05; **p < 0.01; ***p < 0.001). (**h**) DDX3 knockdown repressed primary transcript expressions of tumor-suppressive miRNAs. Primary transcript expressions of miR-200b, miR-200c, miR-122, miR-145, miR-10b, miR-519a and GAPDH in shLuc, shDDX3 #2 and shDDX3 #3 cells were analyzed by qRT-PCR. GAPDH was used as internal control. Fold change of each transcript in shDDX3 #2 and shDDX3 #3 cells was relative to that of shLuc cells. Results were derived from at least three independent experiments, and the error bar indicated ± 1 s.d. of the mean. Statistical analyses were carried out using *t* test (*p < 0.05; **p < 0.01; ***p < 0.001).

To validate the possible association between level of these tumor-suppressive miRNAs and DDX3 in HCC, we next perform quantitative real-time PCR to compare their amount in 39 paired tumor and non-tumor total RNA samples acquired from TLCN. As shown in Fig. 5a–d, DDX3 mRNA expression was positively correlated with levels of tumor-suppressive miR-200b (Spearman r = 0.419, *p* = 0.008), miR-200c (Spearman r = 0.425, *p* = 0.007), miR-122 (Spearman r = 0.530, *p* < 0.001) and miR-145 (Spearman r = 0.374, *p* = 0.019). Dysregulation of miR-10b and miR-519a has been reported in HCC^44^,^45^; therefore, we also examined association between expressions of DDX3 and miR-10b as well as miR-519a. However, no significant correlation of DDX3 transcript with miR-10b (Spearman r = 0.078, *p* = 0.638) or miR-519a (Spearman r = 0.237, *p* = 0.147) level was observed (Fig. 5e,f). Further assessment of positive associations between expressions of DDX3 and those tumor-suppressive miRNAs was performed in shLuc, shDDX3 #2 and shDDX3 #3 HepG2 cells. As shown in Fig. 5g, DDX3 knockdown resulted in the reduction of miR-200b, miR-200c, miR-122 as well as miR-145, but not miR-10b and miR-519a, which was consistent with the results obtained from clinical samples. Thus, our findings suggested a functional role of DDX3 in regulating expression of a subset of tumor-suppressive miRNAs.

Given that several studies indicated the involvement of DDX3 in transcriptional regulation of genes^27^, we then investigated whether DDX3 is involved in transcriptional regulation of the above-mentioned tumor-suppressive miRNAs. As shown in Fig. 5h, primary transcripts of miR-200b, miR-200c, miR-122 and miR-145 in shDDX3 #2 and shDDX3 #3 cells were down-regulated as compared with those of shLuc cells, whereas no obvious differences in miR-10b and miR-519a expressions were observed in these cells. Hence, these results implicated that DDX3 may modulate expressions of these tumor-suppressive miRNAs in a transcriptional manner.

### DDX3 knockdown enhances DNMT3A binding and hypermethylation on promoter regions of miR-200b, miR-200c, miR-122 and miR-145

**Figure 6:**
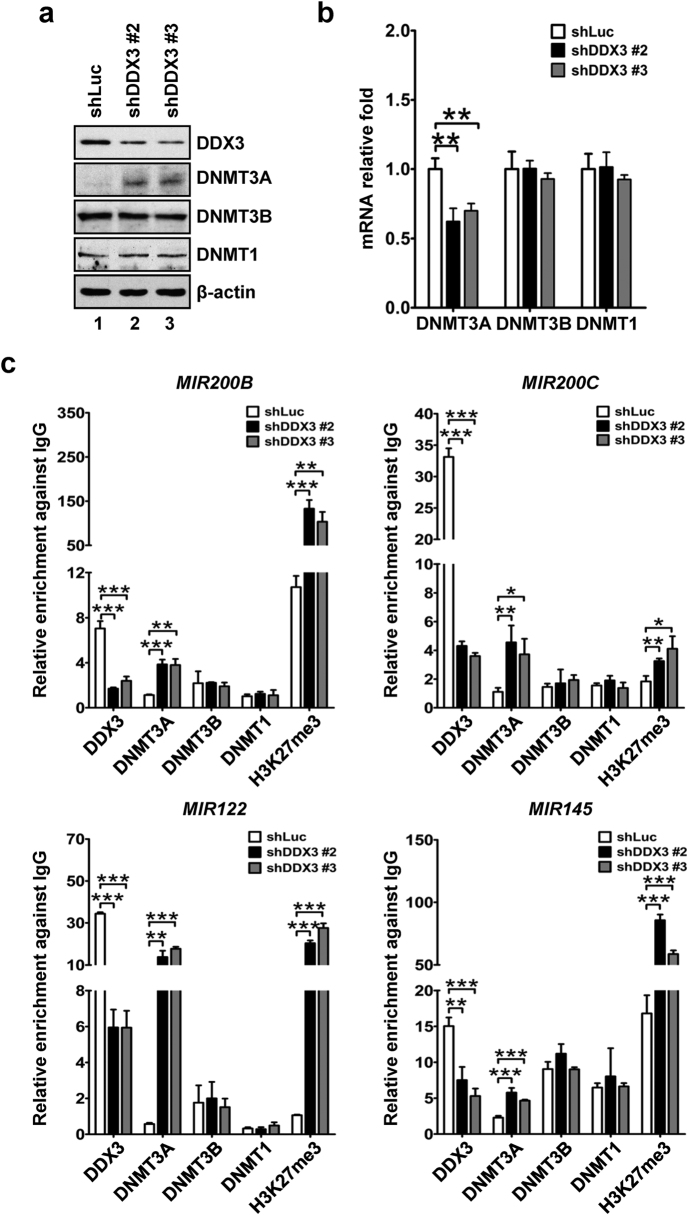
Knockdown of DDX3 enriches DNMT3A binding on promoters of tumor-suppressive miRNAs. (**a**) DDX3 knockdown promoted overexpression of DNMT3A. Cell lysates (50 μg) of shLuc, shDDX3 #2 and shDDX3 #3 cells were analyzed by western blotting with antibodies against DDX3, DNMT3A, DNMT3B, DNMT1 and β-actin. β-actin was used as internal control. (**b**) Suppression of DDX3 is associated with down-regulation of DNMT3A mRNA expression. DNMT3A, DNMT3B, DNMT1 and GAPDH mRNA expressions in shLuc, shDDX3 #2 and shDDX3 #3 cells were analyzed by qRT-PCR. GAPDH was used as internal control. The amount of each transcript in shDDX3 #2 and shDDX3 #3 cells was transformed into fold change relative to that of shLuc cells. (**c**) Knockdown of DDX3 led to reduction of DDX3 binding, enhancement of DNMT3A recruitment and elevation of gene-silencing histone mark H3K27me3 on promoter regions of *MIR200B, MIR200C*, *MIR122* and *MIR145*. shLuc, shDDX3 #2 and shDDX3 #3 cells were subjected to ChIP assay. Binding of DDX3, DNMT3A, DNMT3B, DNMT1 and status of H3K27me3 were expressed as the relative fold change to rabbit IgG binding on the corresponding genomic region, which were normalized with input in individual cell line. All experiments were repeated at least three times, and the error bar indicated ± 1 s.d. of the mean. Statistical analyses were carried out using *t* test (*p < 0.05; **p < 0.01; ***p < 0.001).

As noted, aberrant promoter-associated CpG methylation mediated by deregulation of DNA methyltransferases (DNMTs), including DNMT3A, DNMT3B and DNMT1, usually gives rise to deviant pattern of gene expressions in cancers^20^. It has been shown that promoter DNA hypermethylation attributed to deregulated DNMT expressions is an important mechanism resulting in loss of tumor-suppressive miRNA expressions^21^. For example, studies indicate that promoter hypermethylation of miR-200b, miR-200c, miR-122 and miR-145 suppresses their expressions^23^,^46^,^47^. In our results, we found that knockdown of DDX3 resulted in about 3.4- to 3.8-fold enhancement of DNMT3A expression with no change in DNMT3B and DNMT1 expressions (Fig. 6a). In consistence with protein expression pattern, mRNA level of DNMT 3B or DNMT1 showed no alteration between DDX3-knockdown and control cells. But surprisingly, the expression of DNMT3A mRNA was reduced in shDDX3 #2 and shDDX3 #3 cells compared to that of shLuc cells (Fig. 6b). In order to delineate the involvement of DNMTs in the regulation of miR-200b, miR-200c, miR-122 and miR-145 expressions in DDX3-knockdown cells, binding of DDX3 and DNMTs on promoter regions of these tumor-suppressive miRNAs were analyzed by chromatin immunoprecipitation. As shown in Fig. 6c, DDX3 binding was reduced on promoter regions of *MIR200B* (0.2- to 0.3-fold), *MIR200C* (~0.1-fold), *MIR122* (~0.2-fold) and *MIR145* (0.4- to 0.5-fold) in shDDX3 #2 and shDDX3 #3 cells as compared with that of shLuc cells. In contrast with the reduced binding of DDX3, DNMT3A binding was enriched on promoter regions of *MIR200B* (~3.4-fold), *MIR200C* (3.4- to 4.1-fold), *MIR122* (24.2- to 31.1-fold) and *MIR145* (2.0- to 2.5-fold) in shDDX3 #2 and shDDX3 #3 cells as compared with that of shLuc cells (Fig. 6c). However, DNMT3B or DNMT1 did not exhibit a significant strength as DNMT3A (Fig. 6c). In addition, distribution of gene-silencing histone mark H3K27me3 were elevated on promoter regions of *MIR200B* (9.6- to 12.4-fold), *MIR200C* (1.8- to 2.2-fold), *MIR122* (19.1- to 26.0-fold) and *MIR145* (3.5- to 5.1-fold) in shDDX3 #2 and shDDX3 #3 cells as compared with that of shLuc cells (Fig. 6c). Therefore, depletion of DDX3 augmented DNMT3A binding in association with enhanced H3K27me3 distribution on promoter regions of these tumor-suppressive miRNAs, which reflected transcriptional suppression of these tumor-suppressive miRNAs in DDX3-knockdown cells.

**Figure 7:**
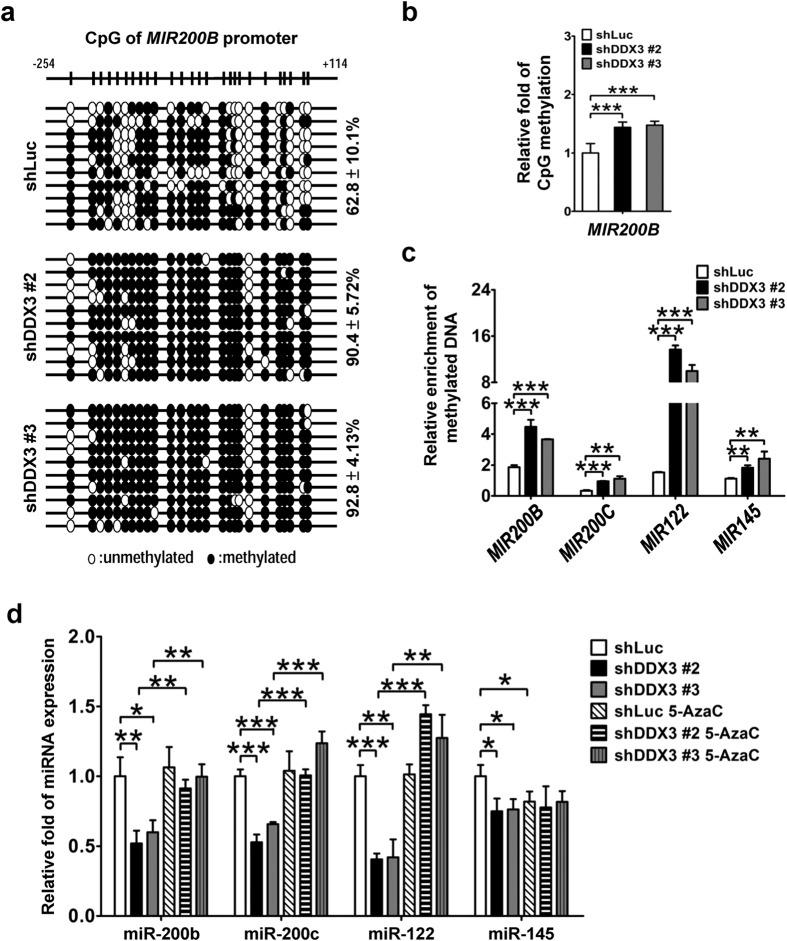
Knockdown of DDX3 results in promoter hypermethylation of tumor-suppressive miRNAs. (**a**) DDX3 knockdown promotes CpG methylation on promoter region of *MIR200B*. DNA fragments for bisulfite sequencing were prepared from shLuc, shDDX3 #2 and shDDX3 #3 cells and analyzed as described in Materials and methods. (**b**) The percentage of *MIR200B* promoter CpG methylation in shDDX3 #2 and shDDX3 #3 cells shown in (**a**) was transformed into fold change relative to that in shLuc cells. (**c**) Cytosine methylation on promoter regions of *MIR200B, MIR200C*, *MIR122* and *MIR145* was enhanced in DDX3-knockdown cells. Genomic DNA extracted from shDDX3 #2 and shDDX3 #3 HepG2 cells was subjected to methylated DNA enrichment assay. The methylation status was expressed as the relative fold change to protein A-magnetic beads binding on the corresponding genomic region, which were normalized with input in individual cell line. (**d**) Treatment with DNMT inhibitor 5-azacytidine (5-AzaC) restored expressions of tumor-suppressive miRNAs in DDX3-knockdown cells. shLuc, shDDX3 #2 and shDDX3 #3 cells were treated with 2 μM 5-azacytidine for 48 h. Expressions of miR-200b, miR-200c, miR-122 and miR-145 and U6 snRNA were analyzed by qRT-PCR. U6 snRNA was used as internal control. Fold change of each transcript in untreated and treated shDDX3 #2 and shDDX3 #3 cells as well as that in treated shLuc cells were relative to that of untreated shLuc cells. All experiments were repeated at least three times, and the error bar indicated ± 1 s.d. of the mean. Statistical analyses were carried out using *t* test (*p < 0.05; **p < 0.01; ***p < 0.001).

To investigate whether transcriptional repressions of *MIR200B, MIR200C*, *MIR122* and *MIR145* are attributed to hypermethylation of their promoter regions in DDX3-knockdown cells, bisulfite sequencing analysis was performed. The percentage of CpG methylation on *MIR200B* promoter region was increased from 62.8% in shLuc cells to 90.4% and 92.8% in shDDX3 #2 and shDDX3 #3 cells, respectively (Fig. 7a), indicating ~1.4-fold enhancement of promoter methylation of *MIR200B* while DDX3 was knockdown (Fig. 7b). As for bisulfite sequencing analysis of *MIR200C*, *MIR122* and *MIR145* promoters, we didn’t collect enough clones to obtain a conclusive result. Alternatively, we performed methylated DNA enrichment assay to assess cytosine methylation level of these tumor-suppressive miRNA promoters (see Materials and methods). In consistent with the results described in Fig. 7a,b, 2.0- to 2.4-fold enrichment of methylated *MIR200B* promoter region was observed in shDDX3 #2 and shDDX3 #3 cells compared to that of shLuc cells (Fig. 7c). Also, enhanced methylated promoter regions of *MIR200C* (2.8- to 3.3-fold), *MIR122* (6.5- to 9.0-fold) and *MIR145* (1.6- to 2.2-fold) in DDX3-knockdown cells suggested enhancements of promoter methylation of these tumor-suppressive miRNAs (Fig. 7c). Thus, these results supported that the reduction of DDX3 contributed to promoter hypermethylation of these tumor-suppressive miRNAs. Additionally, to examine whether reverse methylation status relieves transcriptional repressions of *MIR200B, MIR200C*, *MIR122* and *MIR145* in DDX3-knockdown cells, miRNA expressions in cells were analyzed after treatment with DNMT inhibitor 5-azacytidine for 48 hours. As shown in Fig. 7d, expressions of miR-200b, miR-200c, and miR-122 in shDDX3 #2 and shDDX3 #3 cells were restored to a level close to that of untreated shLuc cells. As for miR-145, 5-azacytidine treatment did not affect its expression in DDX3-knockdown cells, and even led to about 19% of repression in control cells (Fig. 7d). Also noted, the expression pattern of miR-145 in cells with prolonged 5-azacytidine treatment (96 hours) was similar to that observed at 48 hours (Supplementary Figure S3). This phenomenon may be due to 5-azacytidine-induced deregulation of various mechanisms that participate in the regulation of miR-145 expression^48^.

### Overexpression of miR-200b, miR-200c, miR-122 and miR-145 counteracts DDX3 knockdown-induced CSC phenotypes

**Figure 8:**
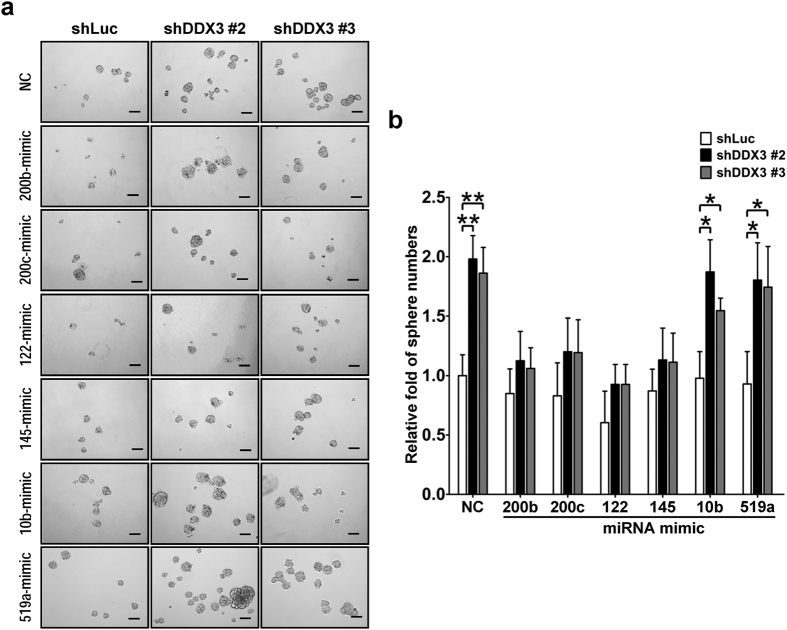
Introduction of tumor-suppressive miRNAs reduces DDX3 knockdown-promoted sphere formation. (**a**) Sphere-forming capability of DDX3-knockdown cells was repressed by tumor-suppressive miRNAs. shLuc, shDDX3 #2 and shDDX3 #3 cells were transfected with negative control (NC) miRNA and specific miRNA mimics of miR-200b, miR-200c, miR-122, miR-145, miR-10b and miR-519a. At 48 h post transfection, cells were subjected to sphere formation assay. Scale bar is equal to 100 μm. (**b**) The number of formed spheres in miRNA-transfected cells described in (**a**) was transformed into fold change relative to that of NC-transfected shLuc cells. Experiments were performed at least three times, and the error bar indicates ± 1 s.d. of the mean. Statistical analyses were carried out using *t* test (*p < 0.05; **p < 0.01).

It has been noted that tumor-suppressive miR-200b, miR-200c, miR-122 and miR-145 are down-regulated in HCC, which are associated with occurrence of CSC traits^10^,^12^,^49^,^50^. As DDX3 knockdown compromised expressions of these tumor-suppressive miRNAs (Fig. 5) and promoted development of CSC traits (Figs 3 and 4), we speculated that these miRNAs might mediate regulation of CSC properties in DDX3-knockdown cells. In such scenario, elevated level of these miRNAs would prevent DDX3 knockdown-induced CSC properties. To test this hypothesis, miRNA mimic of miR-200b, miR-200c, miR-122 and miR-145 was individually transfected into shLuc, shDDX3 #2 and shDDX3 #3 cells, and the transfected cells were subjected to sphere formation assay and EpCAM^+^ population analysis. As shown in Fig. 8, DDX3 knockdown-induced sphere formation was suppressed upon introduction of miRNA mimic of miR-200b, miR-200c, miR-122 or miR-145. In addition, raising level of miR-200b, miR-200c, miR-122 or miR-145 counteracted DDX3 knockdown-mediated enhancement of EpCAM^+^ population (Table 3). Alternatively, when mimic of miR-10b and miR-519a was introduced, no observable change was detected as expected in control and DDX3-knockdown cells. Therefore, these observations suggested that DDX3 repressed the development of CSC properties in liver cells through regulating expression of a subset of tumor-suppressive miRNAs.

## Discussion

DDX3 plays an important role in regulating a variety of cellular events which gives rise to tumorigenesis^27^,^51^. Previous study indicated that DDX3 overexpression facilitates generation of cancer stem cells (CSCs) in lung cancer cells harboring epidermal growth factor receptor (EGFR)-activating mutations^33^. Apart from its oncogenic role, DDX3 also acts as tumor suppressor in several types of cancers^35^,^37^, including HCC. In this study, down-regulation of DDX3 induces stemness gene expressions that confer cells with CSC phenotypes, such as self-renewal, chemoresistance, EMT and migration (Fig. 3). Furthermore, reduction of DDX3 in HCC promotes expansion of CSC populations (Fig. 4), which reflects enhanced tumor-initiating capability (Table 1 and Supplementary Figure S2). Through epigenetic modulation of tumor-suppressive miR-200b, miR-200c, miR-122 and miR-145 transcriptions (Figs 5h, 6c and 7a–c), DDX3 exerts its tumor-suppressive function to prevent the expansion of CSCs in HCC (Fig. 8 and Table 3). Therefore, these studies suggest that DDX3 could be a double-edged sword in regulating generation of CSCs under different cellular context.

**Figure 9:**
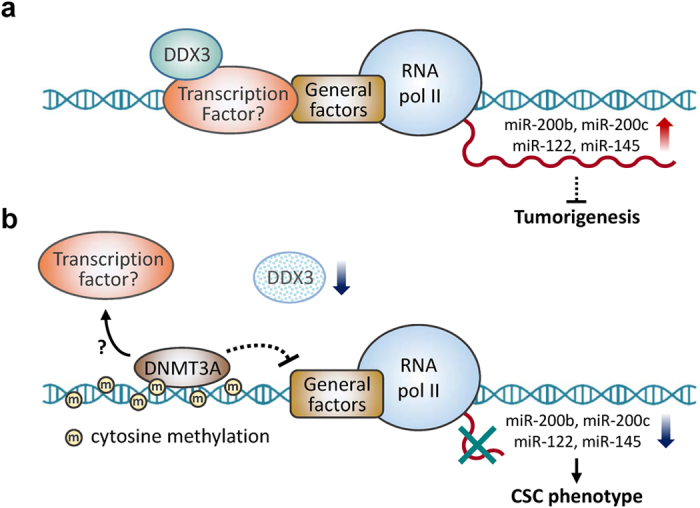
A proposed model illustrates that DDX3 represses stemness by epigenetically modulating tumor-suppressive miRNAs in HCC. (**a**) In physiological condition, DDX3 may interact with transcription factors on promoter regions of a subset of tumor-suppressive miRNA genes, such as *MIR200B, MIR200C*, *MIR122* and *MIR145*, and participate in these miRNA expressions, thereby suppressing CSC phenotypes as well as tumorigenesis in liver. (**b**) Loss of DDX3 may increase the accessibility of DNMT3A to promoter regions of *MIR200B, MIR200C*, *MIR122* and *MIR145*. Enhanced DNMT3A binding on promoter not only stabilizes itself, but also results in hypermethylation of DNA and dissociation of transcription factors. Therefore, expressions of these tumor-suppressive miRNAs are silenced, which induces CSC phenotypes in liver cells.

Accumulating evidence indicates that aberrant expression of miRNAs contributes to tumor initiation, relapse, as well as poor prognosis of patients, and further characterization implicates their potential role in eradication of CSCs in several malignancies, including HCC^52^. For example, restoration of miR-200b, miR-200c and miR-145 in CSCs sensitizes them to chemotherapy, and suppresses CSC phenotypes^8^,^9^,^10^,^52^. Overexpression of miR-122 in CD133^+^ hepatic CSCs compromises their capabilities of chemoresistance and self-renewal^12^. It is clear that individual miRNA could influence a broad spectrum of genes making it a promising therapeutic agent for targeting CSCs^52^. Delineating the important factors in regulating expressions of therapeutic miRNAs might be helpful to improve therapeutic efficacy. Our present study demonstrated that DDX3 knockdown deregulates a list of miRNAs targeting stem cell-like properties-associated genes (Table 2). Subsequent validation of these miRNA candidates showed that knockdown of DDX3 down-regulates transcription of tumor-suppressive miR-200b, miR-200c, miR-122 and miR-145 (Fig. 5h). We found that DDX3 knockdown enhanced protein level and DNA binding of DNMT3A as well as DNA methylation on promoter regions of these tumor-suppressive miRNAs (Figs 6a,c and 7a–c), which results in suppressing transcription of these miRNAs. Therefore, a molecular model for deciphering the role of DDX3 in inhibiting hepatic CSCs induction was proposed (Fig. 9). In viewing that the interaction between DDX3 and transcription factors regulates gene expression^27^, DDX3 may transactivate promoter activity of a subset of tumor-suppressive miRNAs, such as *MIR200B* (Supplementary Figure S4 and Fig. 9a). When DDX3 was depleted, loss of DDX3 increases DNMT3A accessibility to the promoter regions of these tumor-suppressive miRNAs and inhibit their expressions (Fig. 9b). It has been reported that a myriad of transcriptional and post-translational mechanisms participate in regulation of DNMT expressions and protein stability^53^. Recent study manifested that binding of DNMT3A to methylated DNA sequence maintains its protein stability^54^. Therefore, in our case, enhanced DNA binding of DNMT3A may stabilize itself when DDX3 is knockdown (Fig. 6a,c). The reduced expression of DNMT3A mRNA supports this notion (Fig. 6b). Through this molecular mechanism, reduction of DDX3 sustains DNMT3A protein stability and facilitates epigenetic silence of tumor-suppressive miRNA transcriptions to promote CSC phenotypes as well as tumorigenesis (Fig. 9b), which reinforces the tumor suppressor role of DDX3 in HCC.

DDX3 is a multi-functional protein that modulates cellular events through regulation of RNA metabolism, including transcription^27^. For example, association of DDX3 with transcription factor Sp1 synergistically activates transcription of p21^waf1/cip1^ to suppress cell growth^35^, and this interaction also facilitates transcriptional activation of MDM2 to inhibit EMT^37^. In the present study, we found that loss of DDX3 enhances protein level and DNA binding of DNMT3A, which hypermethylates promoter regions of *MIR200B*, *MIR200C*, *MIR122* and *MIR145* (Figs 6a,c and 7a–c). Consequently, knockdown of DDX3 epigenetically suppresses their transcriptions (Fig. 5h). Notably, overexpression of DNMT3A may link to epigenetic silence of tumor suppressors^55^. Here, our results further demonstrated that overexpression of DNMT3A is associated with suppression of PTEN and RASSF1A, but not p53 and RB1, in DDX3-knockdown cells (Supplementary Figure S5). Collectively, DDX3 may impede recruitment of DNMT3A to promoter regions of a group of tumor suppressor genes, including miRNAs (Fig. 6c). In addition to acting as a tumor suppressor, DDX3 also plays an oncogenic role through modulating gene transcriptions. For example, DDX3 binds to E-cadherin promoter to inhibit its expression, and is required for the expression of EMT transcription factor Snail to alter cell morphology, proliferation and migration in breast and cervical cancers^27^,^31^. It has been shown that activation of Snail suppresses transcription of miR-101, which results in promotion of invasion, migration and EMT^56^. Thus, DDX3 might also exert its oncogenic role through modulation of miRNA expressions. Altogether, these findings strengthen the functional role of DDX3 in regulating miRNA transcriptions during tumorigenesis.

It has been noted that the dysregulation of key components of miRNA biogenesis globally affects mature miRNA levels. Also noted, several RNA-binding proteins, including DEAD-box RNA helicases, take part in regulation of miRNA biogenesis^26^. For example, DDX5 and DDX17, two DEAD-box RNA helicases, have been shown to modulate Drosha-mediated processing of pri-miRNAs, suggesting their functional roles in pri-miRNA processing^19^,^26^. However, even though both RNA helicases participate in tumorigenic events of cancer^26^, it is unclear whether they control the tumorigenesis through regulating miRNAs biogenesis. As another accessory protein associated with Drosha complex, DDX3 is thought to be involved in the regulation of miRNA biogenesis^38^. In this study, we identified a list of miRNA candidates affected by knockdown of DDX3 (Table 2) and verified the expressions of tumor-suppressive miR-145, miR-200b, miR-200c and miR-122, but not miR-10b and miR-519a, positively correlated with DDX3 level in HCC clinical samples (Fig. 5a–f). Most importantly, we found that DDX3 knockdown leads to enriched DNMT3A binding as well as hypermethylation on promoter regions of these tumor-suppressive miRNAs (Figs 6c and 7a–c), thereby silencing their transcription (Fig. 5h). In consistence with their tumor-suppressive roles, these tumor-suppressive miRNAs mediate the repression of CSC phenotypes in DDX3-knockdown cells (Fig. 8 and Table 3). Therefore, these findings reveal that DDX3 may exert its tumor suppressor function through epigenetically modulating transcriptions of a subset of tumor-suppressive miRNAs in HCC. It would be interesting to investigate whether DDX3, as a Drosha complex-interacting factor, participates in miRNA biogenesis processes other than transcription.

miR-122, a liver-abundant miRNA, acts as a key regulator in maintaining liver differentiation, function and homeostasis, and its repression is associated with poor differentiation status and predicts poor prognosis of HCC patients^57^. During hepatitis viral infection, miR-122 is a crucial factor in facilitating hepatitis C virus (HCV) infection, but it appears to counteract hepatitis B virus (HBV) replication^57^. In accordance with recent studies, both DDX3 and miR-122 facilitate HCV infection, while they exert inhibitory functions on HBV replication^27^,^57^. We found that miR-122 was most significantly down-regulated among the four tumor-suppressive miRNAs analyzed in DDX3-knockdown HepG2 cells (Fig. 5g), which is consistent with the results that DDX3 knockdown induces most significant DNMT3A recruitment as well as hypermethylation status on promoter region of *MIR122* (Figs 6c and 7c). Moreover, DDX3 suppresses generation of CSCs in HepG2 cells through modulating expression of miR-122 (Fig. 8 and Table 3). These observations suggest the pivotal role of DDX3 in maintaining differentiation of liver cells and preventing tumorigenesis in liver. In addition, our previous study indicates that reduction of DDX3 expression is correlated with infection of HBV but not HCV in HCC patients^34^. Therefore, in this study, we establish positive correlation between DDX3 and miR-122 (Fig. 5c,g), which may delineate the regulatory mechanism during HBV infection as well as hepatic tumorigenesis.

Cancer stem cells (CSCs) are responsible for the poor prognosis of patients, and development of therapeutic strategies against these subpopulations would be beneficial to survival of patients^3^. In the last decade, the potential of DDX3 as a therapeutic target has been assessed in different malignancies^51^. For example, inhibitors targeting activities or RNA interference-mediated suppression of DDX3 circumvent its tumorigenic potentials^51^. Studies also demonstrate that overexpression of DDX3 in human lung cancer cells induces CSC phenotypes^33^, and its overexpression in murine CD133^+^ melanoma cells serves as an immunogenic target for eradication of CSCs^32^. To date, most studies are focusing on DDX3 overexpression as therapeutic target. However, in this study, our findings suggest that the reduction of DDX3 in HCC subsequently promotes expansion of CSC populations (Fig. 4), and predicts poor prognosis of patients (Fig. 1b). Through epigenetic modulation of a subset of tumor-suppressive miRNA transcriptions (Figs 5h, 6c and 7a–c), DDX3 functions as a tumor suppressor to inhibit the expansion of CSCs in HCC (Fig. 8 and Table 3). Taken together, our present study illustrates a novel mechanism that DDX3 epigenetically regulates a subset of tumor-suppressive miRNAs expressions to inhibit the induction of stemness in HCC (Fig. 9), which may provide helpful insights into the potential of DDX3 as a therapeutic target for elimination of hepatic CSCs.

## Materials and methods

### Ethics statements

Total RNA and frozen tissues from 39 paired HCC tumor and non-tumor tissue samples were provided by Taiwan Liver Cancer Network (TLCN), Taiwan. The clinicopathological information of participating patients (all anonymous) is summarized in Supplementary Table S1. Informed consent was obtained from all patients, and research ethics was approved by the Institutional Review Board, Taipei Veterans General Hospital, Taiwan (VGHIRB No.: 2012-05-042BY). All experiments were performed in accordance with relevant guidelines and regulations approved by the committee.

The animal experiments were approved by the Institutional Animal Care and Use Committee (IACUC) of National Yang-Ming University, Taiwan (IACUC No.: 1040627). The animal care and experimental procedures were carried out in accordance with the Guidelines of the IACUC of National Yang-Ming University, Taiwan.

### Analysis of published clinical microarray data and TissueScan liver cancer cDNA array

An *in silico* analysis on DDX3 expression of *Ye* Liver clinical microarray dataset^39^ in a public cancer microarray database *Oncomine* (http://www.oncomine.org) was performed. Statistical outlier analysis was carried out on the database. Survival probability of patients in this dataset was analyzed by Kaplan-Meier estimator. TissueScan Liver Cancer cDNA Array was purchased from OriGene Technologies (Rockville, MD, USA). The clinicopathological information of patients and method of analyzing gene expression for the cDNA array is available on the manufacturer’s website (http://www.origene.com). Only results from normal and HCC tissues were subjected to DDX3 expression analysis.

### Extraction of total RNA, reverse transcription and quantitative real-time polymerase chain reaction (qRT-PCR)

Extraction of total RNA from cell lines was performed using TRI Reagent (Sigma, St. Louis, MO, USA) following manufacturer’s manual. cDNA of mRNA or miRNA was synthesized by First strand cDNA synthesis kit (Thermo Scientific, Pittsburgh, PA, USA) or TaqMan MicroRNA Reverse Transcription Kit (Applied Biosystems, Foster City, CA, USA), respectively. qRT-PCR analysis of mRNA expression was performed on StepOnePlus Real-Time PCR Systems (Applied Biosystems) using KAPA SYBR FAST qPCR Kit (KAPA Biosystems, Wilmington, MA, USA) with primers listed in Supplementary Table S2. miRNA expression was analyzed by qRT-PCR using TaqMan Universal Master Mix II with custom TaqMan probes and primers (Applied Biosystems) whose Assay ID is listed in Supplementary Table S3.

### Preparation of total protein from cell lines and HCC specimen

Extraction of total protein from cell lines or frozen tissues of TLCN HCC patients was performed as previously described^34^.

### Antibodies

Anti-DDX3 antibodies were generated as described previously^34^,^35^. Antibodies against Nanog (#3580), Oct4 (#2840), c-Myc (#9402), Bmi1 (#2830) and CK19 (#4558) were purchased from Cell Signaling Technology (Danvers, MA, USA). Anti-Sox2 (GTX627404), anti-KLF4 (GTX101508), anti-DNMT3A (GTX116011), anti-DNMT3B (GTX62171) and anti-DNMT1 (GTX62550) antibodies were purchased from GeneTex (Irvine, CA, USA). Anti-β-actin antibodies were purchased from Sigma. Antibodies against E-cadherin (610181) and fibronectin (610077) were purchased from BD biosciences (Franklin Lakes, NJ, USA). Phycoerythrin (PE)-conjugated anti-CD133 (130-090-853), anti-CD13 (130-103-733), anti-EpCAM (130-091-253) and anti-CD90 (130-095-400) antibodies were purchased from Miltenyi Biotec (Bergisch Gladbach, Germany). H3K27me3 antibody (ABE44) was purchased from Millipore (Cambridge, MA, USA).

### Cell culture, transfection, lentivirus production and establishment of DDX3-knockdown HepG2 cells

HepG2, Hep3B, HuH-7, SK-Hep-1 and HEK293T were grown as previously described^34^,^35^. Plasmids for short hairpin RNA (shRNA) lentivirus production, including pLKO.1-based shRNA vectors, pCMV-ΔR8.91 (*gag*, *pol* and *rev* expressing plasmid) and pMD.G (vesicular stomatitis virus glycoprotein expressing plasmid), were obtained from the National RNAi Core Facility, Academia Sinica, Taiwan. The targeting sequences of shRNAs were as follows: 5′-CTTCGAAATGTCCGTTCGGTT-3′ for shLuc, 5′-CGGAGTGATTACGATGGCATT-3′ for shDDX3 #2 and 5′-CGTAGAATAGTCGAACAAGAT-3′ for shDDX3 #3. Lentivirus production in HEK293T cells was performed according to the protocol suggested by the RNAi Core. HepG2 cells were infected with lentivirus at a multiplicity of infection (M.O.I.) of 1 in the presence of 10 μg/ml polybrene (Sigma) for 16 h and selected using 2 μg/ml of puromycin (Sigma) for one week. Stable control (shLuc) and DDX3-knockdown (shDDX3 #2 and shDDX3 #3) HepG2 cells were obtained after verification of DDX3 knockdown efficiency by western blotting and qRT-PCR.

Plasmid pcDNA3-SRα/FLAG or pcDNA3-SRα/FLAG-DDX3^35^ (15 μg) was transfected into SK-Hep-1 cells (10^6^ cells/10-cm dish) using TransIT-LT1 transfection reagent (45 μl) according to the manufacturer’s instruction (Mirus Bio LLC, Madison, WI, USA). mirVana miRNA mimics (100 pmole each), including negative control miRNA and specific miRNA mimics of miR-200b, miR-200c, miR-122, miR-145, miR-10b and miR-519a (Applied Biosystems), were individually transfected into shLuc, shDDX3 #2 and shDDX3 #3 cells (3 × 10^5^ cells/well of a 6-well plate) using Lipofectamine 2000 (5 μl) following manufacturer’s manual (Invitrogen, Karlsruhe, Germany).

### Sphere formation assay

Single-cell suspension of 1 × 10^3^ cells were subjected to sphere formation assay and quantified as previously described^58^. Images of formed spheres were captured under Leica DMI 400B inverted microscope (Leica, Wetzlar, Germany) at 100X magnification.

### Anti-cancer drug treatment

shLuc, shDDX3 #2 and shDDX3 #3 cells (1 × 10^4^ cells/well in a 96-well plate) were treated with different concentrations of anti-cancer drugs doxorubicin (0, 0.125, 0.25 and 0.5 μg/ml) or 5-fluorouracil (0, 2, 4 and 8 μg/ml) for 72 h. Cell viability was assessed by their ability to transform 3-[4,5-di-methylthiazol-2-yl]-2,5-diphenyltetrazolium bromide (MTT) into purple formazan and absorbance was determined at 550 nm.

### Migration assay

shLuc, shDDX3 #2 or shDDX3 #3 cells (2.5 × 10^5^ cells each) were subjected to migration assay as previously described^22^. After 48 h incubation, cells were fixed with methanol and stained with Giemsa (Sigma), and the migrated cells were photographed using an inverted microscope Axiovert S100 (Carl Zeiss, Oberkochen, Germany) under 10X objective lens equipped with NIKON CoolPix 995 digital camera (Nikon, Tokyo, Japan).

### Flow cytometry and isolation of EpCAM^+^ cells

shLuc, shDDX3 #2 and shDDX3 #3 cells (1 × 10^6^ cells each) were stained with phycoerythrin (PE)-conjugated anti-CD133, anti-CD13, anti-EpCAM, anti-CD90 or isotype control antibodies according to the manufacturer’s instruction (Miltenyi Biotec). Cells were analyzed by fluorescence-activated cell sorting (FACS) on FACSCalibur flow cytometer (BD Biosciences). Isolation of EpCAM^+^ and its counterpart HepG2 cells was performed on a BD FACSAria cell sorting system (BD Biosciences).

### Xenograft transplantation

Various numbers of shLuc, shDDX3 #2 and shDDX3 #3 cells were suspended in phosphate buffered saline and transplanted to 5-week old male NOD/SCID mice (BioLASCO Taiwan, Taipei, Taiwan). Cells were implanted subcutaneously at the flank region of back limbs. Tumor initiation was monitored and recorded every 2 weeks.

### Small RNA library preparation and next-generation sequencing (NGS)

Total RNA extracted from shLuc, shDDX3 #2 and shDDX3 #3 cells were subjected to Agilent 2200 TapeStation-R6K assay for quality analysis. All RNA samples were highly intact with RNA Integrity Number greater than 8.5, which were cleaned and enriched the small RNA fraction by RNA Clean & Concentrator-5 column (Zymo Research, Irvine, CA, USA). Small RNA enriched from 6 μg of total RNA was used for library construction using TruSeq Small RNA Library Preparation Kit (Illumina Inc., Hayward, CA, USA) according to the manufacturer’s instructions. The concentration of libraries was determined by KAPA Library Quantification Kit for NGS (KAPA Biosystems). Libraries (2 nM) were sequenced on MiSeq (Illumina Inc.) by single end sequencing with a 50 bp read length.

### Bioinformatics for miRNA expression profiles

Raw sequencing reads were trimmed and quality pre-processed as follows. First of all, the Illumina small RNA 3′ adapter sequence 5′-TGGAATTCTCGGGTGCCAAGG-3′ was removed. The reads were then trimmed according to their quality values based on Phred quality score. Nucleotides with high-quality base calling (Phred quality score ≥ 20, that represents 99% accuracy of a base call) were included, and small RNA reads that were longer than 18 nucleotides were retained. The small RNA distribution and microRNA quality in the sequencing data were verified by using ncPRO-seq^59^ package (version 1.6.1) to confirm the read distribution in the reference genome. Only reads that mapped a maximum of one mismatches and 20 locations in the genome were used (bowtie v1.1.2 parameter: -v1 -a -m20 –best –strata –nomaqround -f -y). Five databases were employed for annotation: the UCSC reference genome (hg19), miRBase v21, UCSC refGene, RFam v11.0, and UCSC repeatMasks (hg19). To quantify miRNA expression profiles, we used miRDeep2^60^ package (version 2.0.0.5) and employed the same bowtie parameter as ncPRO-seq. To normalize miRNA expression across different samples, the reads count in each sample was normalized using reads per million (RPM). In order to filter very low expression miRNA, multiplying of the expression value in two compared samples was more than 10 RPM. The miRNAs were identified as up- or down-regulated miRNA if the fold change was greater than 1 or less than 1, respectively.

### Chromatin immunoprecipitation (ChIP)-qRT-PCR

ChIP-qRT-PCR assay was performed as previously described^58^. DNA fragments derived from decross-linked protein-DNA complex were extracted and purified by ChIP DNA Clean & Concentrator (Zymo Research). Gene specific primer sets for qRT-PCR analysis are summarized in Supplementary Table S4.

### Bisulfite sequencing

Bisulfite sequencing of *MIR200B* promoter region was performed as previously described^24^. Genomic DNA was extracted and bisulfite converted using QIAamp DNA Mini Kit (QIAGEN, Valencia, CA, USA) and EZ DNA Methylation-Lightning Kit (Zymo Research), respectively. The primer set for the sequencing region (254-nt upstream and 114-nt downstream from the transcription start site) was as follows: Forward: GGTTTTATAGAAGTTTTTTTATTTTGGTTT; Reverse: CACAAAAAATCAATTCAAACCTACACAAA). Underlined nucleotides in Forward primer or Reverse primer were transformed from C of original sense and G of original anti-sense sequences, respectively. The PCR product was extracted and purified using GFX PCR DNA and Gel Band Purification Kit (GE Healthcare, Chicago, IL, USA), followed by cloning into pGEM-T Easy vector (Promega, Madison, WI, USA). At least 10 separate clones for each cell line were collected for DNA sequence analysis.

### Methylated DNA enrichment assay

Genomic DNA was extracted and fragmented using QIAamp DNA Mini Kit (QIAGEN) and Bioruptor Standard Sonication System (Diagenode Inc., Seraing, Belgium), respectively. Methylated DNA fragments were enriched by EpiMark Methylated DNA Enrichment Kit (New England BioLabs Inc., Ipswich, MA, USA) according to manufacturer’s instruction. Gene specific primer sets for qRT-PCR analysis are identical to ChIP-qRT-PCR analysis, which are summarized in Supplementary Table S4.

### 5-azacytidine treatment

shLuc, shDDX3 #2 and shDDX3 #3 cells (5 × 10^5^ cells/well in a 6-well plate) were treated with 2 μM 5-azacytidine (Sigma) for 48 h. Expressions of mature miR-200b, miR-200c, miR-122 and miR-145 were detected and analyzed as described above.

## Additional Information

**How to cite this article**: Li, H.-K. *et al*. DDX3 Represses Stemness by Epigenetically Modulating Tumor-suppressive miRNAs in Hepatocellular Carcinoma. *Sci. Rep.* **6**, 28637; doi: 10.1038/srep28637 (2016).

## Supplementary Material

Supplementary Information

## Figures and Tables

**Table 1:** Tumor-initiating ability of DDX3-knockdwon cells in NOD/SCID xenograft transplant model.

Number of injected cells	Tumorigenic incidence			Latency (weeks)
	shLuc	shDDX3 #2	shDDX3 #3	
1.0 × 10^5^	1/12 (8.3%)*	2/9 (22.2%)	4/9 (44.4%)	2
5.0 × 10^4^	0/8 (0.0%)	1/6 (16.7%)	2/6 (33.3%)	2
1.0 × 10^4^	0/4 (0.0%)	0/3 (0.0%)	0/3 (0.0%)	–
1.0 × 10^3^	0/4 (0.0%)	0/3 (0.0%)	0/3 (0.0%)	–

Note: Tumor initiation was monitored for 10 weeks after implantation.

^*^Delayed tumor formation (6 weeks) was observed compared with tumors derived from DDX3-knockdown cells.

**Table 2:** Functional classification of miRNA candidates affected by DDX3 knockdown in HepG2 cells.

Stem cell-like properties	microRNA	Target gene	PMID	Expression (shDDX3/shLuc)
Self-renewal	miR-200b	BMI1	21840484	Down-regulation
	miR-145	SOX2	19409607	Down-regulation
		KLF4	19409607	
		c-MYC	19202026	
		NANOG	21092188	
		OCT4	24240684	
	miR-204	SOX4	23204299	Down-regulation
	miR-33b	SALL4	25919570	Down-regulation
	miR-1246	CCNG2	25117811	Up-regulation
Differentiation	let-7e	WNT1	19398721	Down-regulation
	miR-200b	WNT1	23851184	Down-regulation
	miR-1275	CLDN11	22736761	Down-regulation
	miR-181a/b/c/d	GATA6	19585654	Up-regulation
		CDX2	19585654	
	miR-92b	C/EBPβ	23936298	Up-regulation
	miR-27a	ZBTB10	23752185	Up-regulation
Chemoresistance	miR-30e	ABL	25305453	Down-regulation
	miR-1246	CCNG2	25117811	Up-regulation
Motility/EMT/invasion	miR-200b	ZEB1	24691972	Down-regulation
	miR-483	CKB	25601461	Down-regulation
	miR-204	EPHB2	23204299	Down-regulation
	miR-145	HMGA2	25444913	Down-regulation
	miR-33b	HMGA2	25919570	Down-regulation
		TWIST1	25919570	
	miR-30a	MTDH	23851509	Up-regulation

**Table 3:** EpCAM^ + ^population in DDX3-knockdown cells was reduced by tumor-suppressive miRNAs.

	% of EpCAM^+^ cells		
	shLuc	shDDX3 #2	shDDX3 #3
NC	1.56 ± 0.15	2.66 ± 0.35**	2.59 ± 0.37**
miR-200b	1.03 ± 0.33	1.08 ± 0.35	1.67 ± 0.33
miR-200c	0.91 ± 0.26	0.84 ± 0.32	0.94 ± 0.22
miR-122	0.77 ± 0.15	0.95 ± 0.17	0.70 ± 0.17
miR-145	1.16 ± 0.27	0.93 ± 0.17	1.30 ± 0.33
miR-10b	1.77 ± 0.39	2.41 ± 0.35	2.17 ± 0.14
miR-519a	1.50 ± 0.54	2.41 ± 0.23	2.60 ± 0.33*

Note: Experiments were performed at least three times, and results was presented as mean ± 1 s.d. Statistical analyses were carried out using *t* test to compare EpCAM^+^ cells in shDDX3 #2 or shDDX3 #3 cells relative to that in shLuc cells (*p < 0.05; **p < 0.01).
