# Supplementary material for: DDX3 Represses Stemness by Epigenetically Modulating Tumor-suppressive miRNAs in Hepatocellular Carcinoma
Source: Sci Rep. 2016 Jun 27;6:28637. doi: 10.1038/srep28637 (PMC4921922; doi:10.1038/srep28637)
Supplement: Supplementary Information [file srep28637-s1.pdf]

Scientific Reports
Supplementary information

DDX3 Represses Stemness by Epigenetically Modulating Tumor-suppressive miRNAs in Hepatocellular Carcinoma

Hao-Kang Li, Ru-Tsun Mai, Hsien-Da Huang, Chih-Hung Chou, Yi-An Chang,
Yao-Wen Chang, Li-Ru You, Chun-Ming Chen and Yan-Hwa Wu Lee

Supplementary Figures S1-S5, Supplementary Method and Supplementary Tables S1-S4

Supplementary Figure S1 Li *et al.*

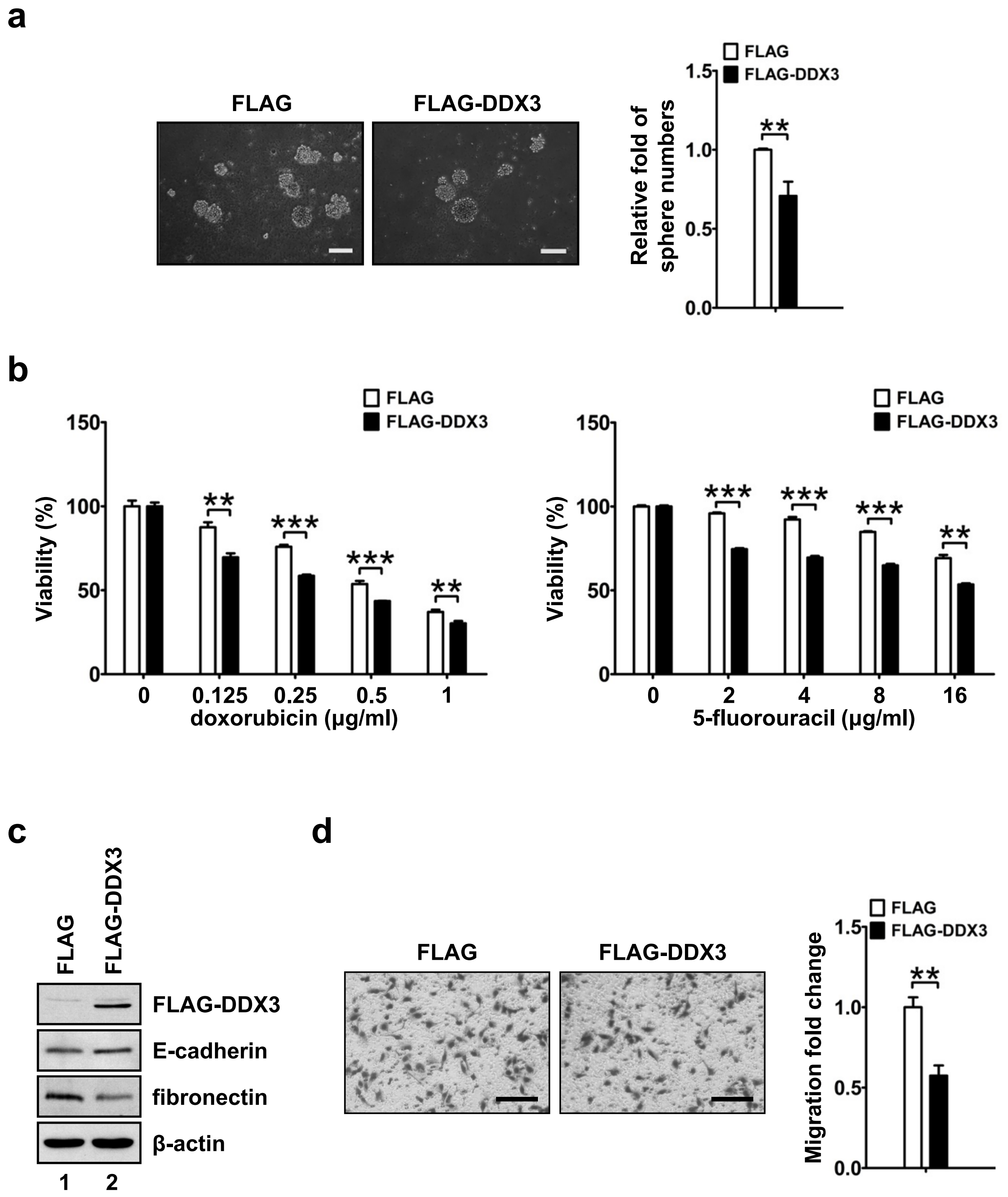

Supplementary Figure S2

Li *et al.*

a

Tag No.	Injection site	Type of injected cells	Number of injected cells	Tumor development
**#327**	1	shLuc	$1x10^5$	
	2	shDDX3 #2	$1x10^5$	
**#328**	3	shLuc	$1x10^5$	
	4	shDDX3 #2	$1x10^5$	
**#329**	5	shLuc	$1x10^5$	
	6	shDDX3 #3	$1x10^5$	Yes
**#330**	7	shLuc	$1x10^5$	
	8	shDDX3 #3	$1x10^5$	
**#331**	9	shDDX3 #2	$1x10^5$	Yes
	10	shDDX3 #3	$1x10^5$	
**#2521**	11	shLuc	$1x10^5$	
	12	shDDX3 #2	$1x10^5$	Yes
**#2522**	13	shLuc	$1x10^5$	
	14	shDDX3 #2	$1x10^5$	
**#2523**	15	shLuc	$1x10^5$	
	16	shDDX3 #2	$1x10^5$	
**#2524**	17	shLuc	$1x10^5$	
	18	shDDX3 #3	$1x10^5$	Yes
**#2525**	19	shLuc	$1x10^5$	
	20	shDDX3 #3	$1x10^5$	
**#2526**	21	shLuc	$1x10^5$	Yes
	22	shDDX3 #3	$1x10^5$	Yes
**#2527**	23	shDDX3 #2	$1x10^5$	
	24	shDDX3 #3	$1x10^5$	
**#2528**	25	shDDX3 #2	$1x10^5$	
	26	shDDX3 #3	$1x10^5$	Yes
**#2529**	27	shDDX3 #2	$1x10^5$	
	28	shDDX3 #3	$1x10^5$	
**#2530**	29	shLuc	$1x10^5$	
	30	shLuc	$1x10^5$	
**#2511**	31	shLuc	$5x10^4$	
	32	shDDX3 #2	$5x10^4$	
**#2512**	33	shLuc	$5x10^4$	
	34	shDDX3 #2	$5x10^4$	Yes
**#2513**	35	shLuc	$5x10^4$	
	36	shDDX3 #2	$5x10^4$	
**#2514**	37	shLuc	$5x10^4$	
	38	shDDX3 #3	$5x10^4$	
**#2515**	39	shLuc	$5x10^4$	
	40	shDDX3 #3	$5x10^4$	
**#2516**	41	shLuc	$5x10^4$	
	42	shDDX3 #3	$5x10^4$	Yes
**#2517**	43	shDDX3 #2	$5x10^4$	
	44	shDDX3 #3	$5x10^4$	Yes
**#2518**	45	shDDX3 #2	$5x10^4$	
	46	shDDX3 #3	$5x10^4$	
**#2519**	47	shDDX3 #2	$5x10^4$	
	48	shDDX3 #3	$5x10^4$	
**#2520**	49	shLuc	$5x10^4$	
	50	shLuc	$5x10^4$	

b

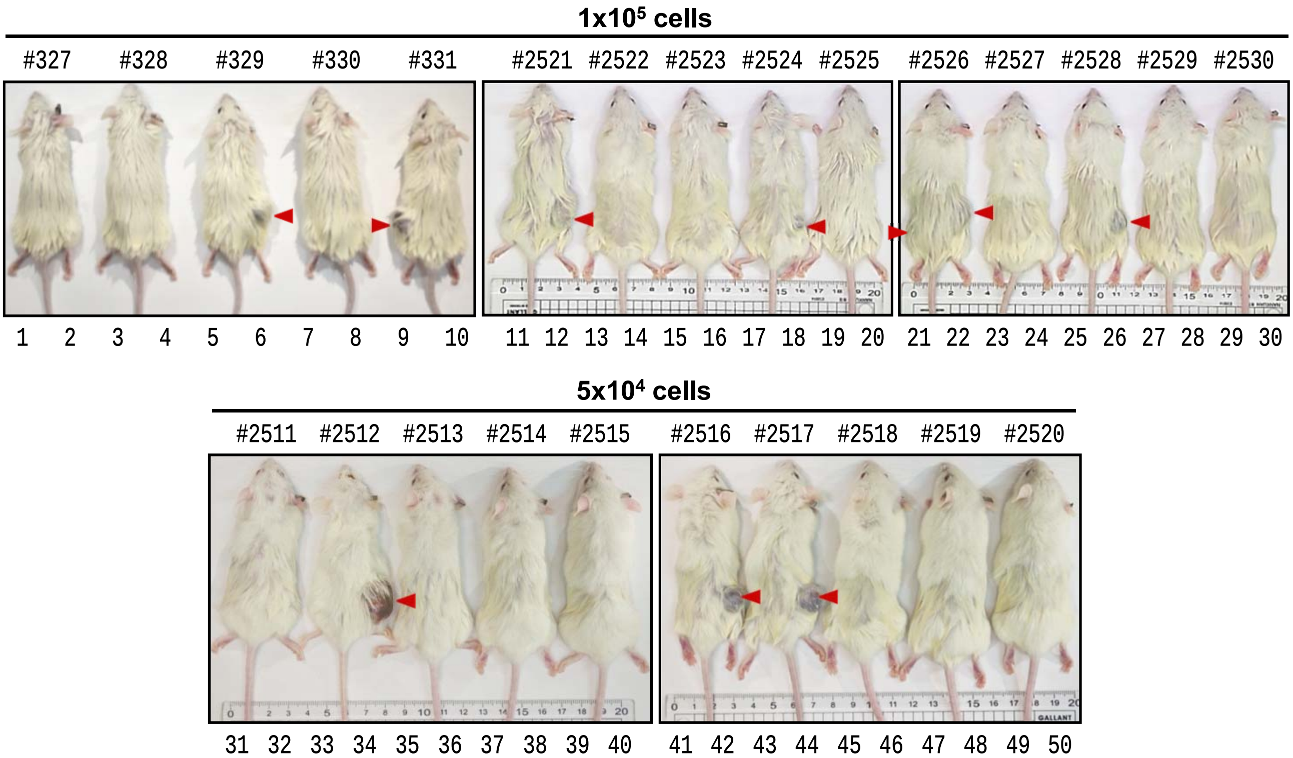
1x10^5 cells
#327 #328 #329 #330 #331
#2521 #2522 #2523 #2524 #2525
#2526 #2527 #2528 #2529 #2530
1 2 3 4 5 6 7 8 9 10 11 12 13 14 15 16 17 18 19 20 21 22 23 24 25 26 27 28 29 30
5x10^4 cells
#2511 #2512 #2513 #2514 #2515
#2516 #2517 #2518 #2519 #2520
31 32 33 34 35 36 37 38 39 40 41 42 43 44 45 46 47 48 49 50

c

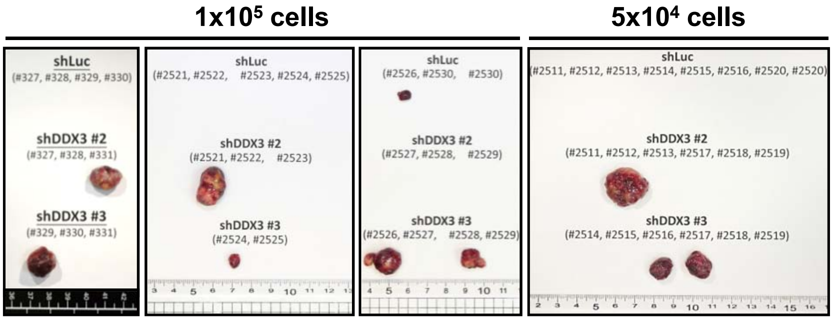
1x10^5 cells
5x10^4 cells
shLuc
(#327, #328, #329, #330)
shDDX3 #2
(#327, #328, #331)
shDDX3 #3
(#329, #330, #331)
shLuc
(#2521, #2522, #2523, #2524, #2525)
shDDX3 #2
(#2521, #2522, #2523)
shDDX3 #3
(#2524, #2525)
shLuc
(#2526, #2530, #2530)
shDDX3 #2
(#2527, #2528, #2529)
shDDX3 #3
(#2526, #2527, #2528, #2529)
shLuc
(#2511, #2512, #2513, #2514, #2515, #2516, #2520, #2520)
shDDX3 #2
(#2511, #2512, #2513, #2517, #2518, #2519)
shDDX3 #3
(#2514, #2515, #2516, #2517, #2518, #2519)

d

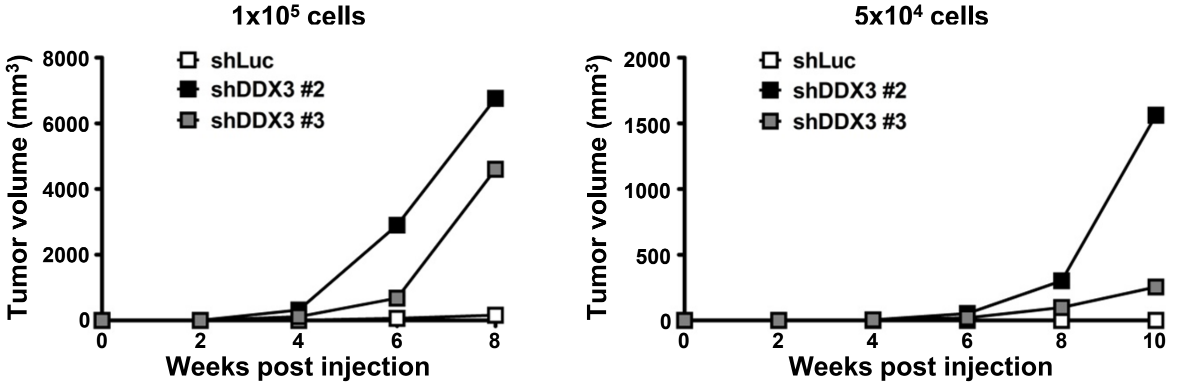
1x10^5 cells
Tumor volume (mm^3)
8000
6000
4000
2000
0
shLuc
shDDX3 #2
shDDX3 #3
0 2 4 6 8
Weeks post injection
5x10^4 cells
Tumor volume (mm^3)
2000
1500
1000
500
0
shLuc
shDDX3 #2
shDDX3 #3
0 2 4 6 8 10
Weeks post injection

Supplementary Figure S3 Li *et al.*

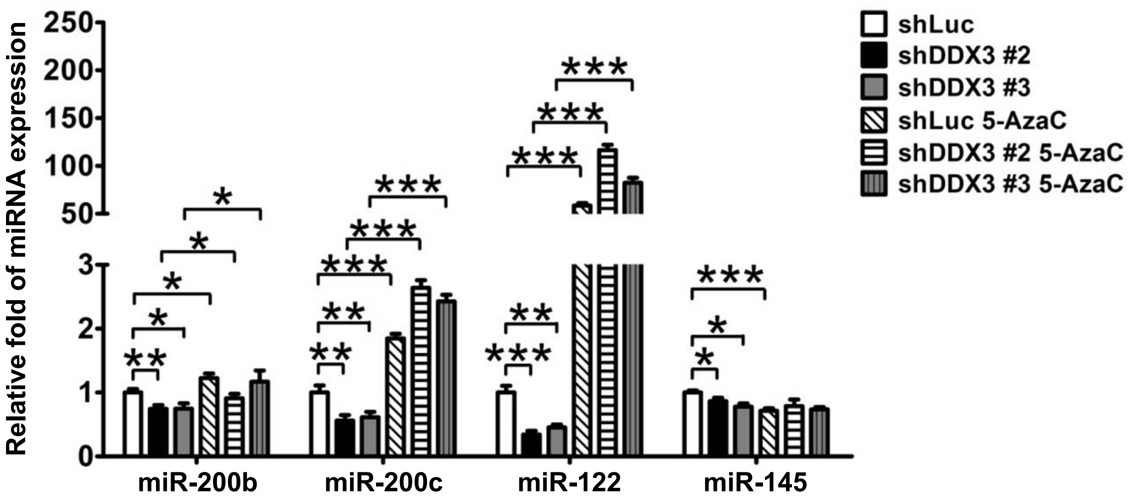

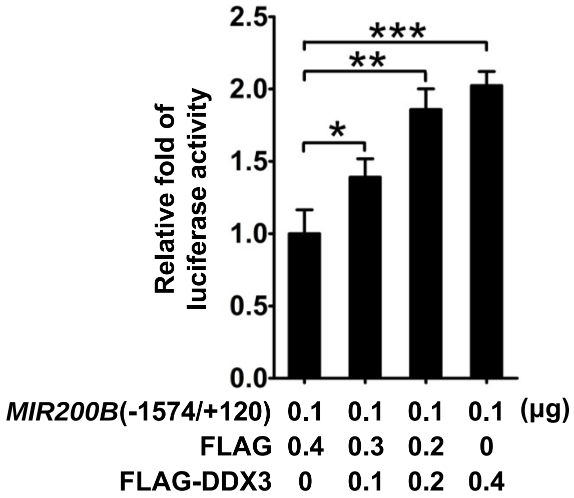
Relative fold of luciferase activity
2.5
2.0
1.5
1.0
0.5
0.0
*
**
***
MIR200B(-1574/+120) 0.1 0.1 0.1 0.1 (μg)
FLAG 0.4 0.3 0.2 0
FLAG-DDX3 0 0.1 0.2 0.4

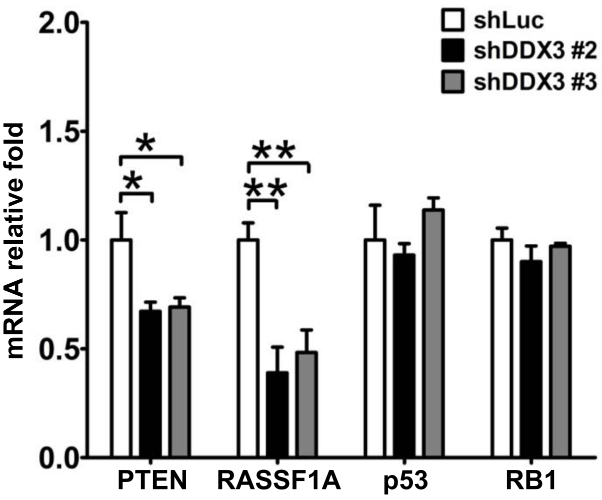
shLuc
shDDX3 #2
shDDX3 #3
mRNA relative fold
2.0
1.5
1.0
0.5
0.0
*
*
**
**
PTEN
RASSF1A
p53
RB1

Supplementary Figure Legends

Supplementary Figure S1. DDX3 inhibits cell capabilities of self-renewal, chemoresistance, EMT and migration. (**a**) DDX3 overexpression repressed self-renewal capability. SK-Hep-1 cells were transfected with plasmid pcDNA3-SRα/FLAG or pcDNA3-SRα/FLAG-DDX3. At 48 h post transfection, cells (1×10^3 cells/well in a 6-well plate) were subjected to sphere formation assay and images of formed sphere were captured. Scale bar represents 100 µm. The numbers of spheres in FLAG-DDX3-expressing cells were transformed into fold change relative to that of vector control cells. (**b**) Up-regulation of DDX3 sensitized cells to conventional anti-cancer drugs treatment. Plasmid pcDNA3-SRα/FLAG or pcDNA3-SRα/FLAG-DDX3 was transfected into SK-Hep-1 cells. At 48 h post transfection, cells (7×10^3 cells/well in a 96-well plate) were treated with different concentrations of doxorubicin (0, 0.125, 0.25, 0.5 and 1 µg/ml; left panel) or 5-fluorouracil (0, 2, 4, 8 and 16 µg/ml; right panel) for 48 h, and cell viability was determined by MTT assay. Formazan absorbance at 550 nm of untreated cells was arbitrarily set as 100% viable. Cell viability at each drug concentration was relative to that of corresponding untreated cells. (**c**) DDX3 overexpression suppressed EMT. SK-Hep-1 cells were transfected with plasmid pcDNA3-SRα/FLAG or pcDNA3-SRα/FLAG-DDX3. At 48 h post transfection, cell lysates (50 µg/each) were analyzed by western blotting with antibodies against FLAG epitope, E-cadherin, fibronectin and β-actin. (**d**) Overexpression of DDX3 inhibited cell migratory ability. Plasmid pcDNA3-SRα/FLAG or pcDNA3-SRα/FLAG-DDX3 was transfected into SK-Hep-1 cells. At 48 h post transfection, cells (1×10^5 cells each) were subjected to migration assay for 12 h and images of Giemsa-stained migrated cells were captured. Scale bar represents 100 µm. The number of migrated cells in FLAG-DDX3-expressing cells was relative to that of vector control cells. All experiments were repeated at least three times, and the error bar indicated ±1 s.d. of the mean. Statistical analyses were carried out using *t* test (**, $p < 0.01$; ***, $p < 0.001$).

Supplementary Figure S2. DDX3 knockdown enhances tumorigenesis. (**a**) Tumors were derived from 1×10^5 or 5×10^4 of shLuc, shDDX3 #2 and shDDX3 #3 HepG2 cells subcutaneously transplanted in 5-week-old NOD/SCID mice. Injection sites at the flank region of left or right back limb in dorsal view of mice were designated from 1 to 50. Cell type, cell number and tumor development of injection sites were summarized. (**b**) Images of mice were shown at sacrifice (8 weeks for 1×10^5 cells and 10 weeks for 5×10^4 cells after injection). Red arrowheads indicated the positions of subcutaneous tumor tissues. (**c**) Images of dissected tumor tissues as well as the corresponding type of injected cell and tag number of mice were shown. (**d**) Tumor volumes were monitored every 2 weeks. Tumor volume was determined by measuring length (L) and width (W) with a caliper and was calculated according to the following formula: tumor volume = $L\times W^2\times0.5$.

Supplementary Figure S3. Prolonged 5-azacytidine treatment further restored expressions of tumor-suppressive miRNAs in DDX3-knockdown cells. shLuc, shDDX3 #2 and shDDX3 #3 cells (5×10^5 cells/well in a 6-well plate) were treated with 2 μM 5-azacytidine for 96 h. Expressions of miR-200b, miR-200c, miR-122 and miR-145 and U6 snRNA were analyzed by qRT-PCR. U6 snRNA was used as internal control. Fold change of each transcript in untreated and treated shDDX3 #2 and shDDX3 #3 cells as well as that in treated shLuc cells were relative to that of untreated shLuc cells. Results were derived from at least three independent experiments, and the error bar indicated ±1 s.d. of the mean. Statistical analyses were carried out using *t* test (*, $p < 0.05$; **, $p < 0.01$; ***, $p < 0.001$).

Supplementary Figure S4. DDX3 activates *MIR200B* promoter activity. *MIR200B* promoter-driven luciferase reporter (0.1 μg) and increasing amount (0, 0.1, 0.2 and 0.4 μg) of plasmid pcDNA3-SRα/FLAG-DDX3 were cotransfected into HepG2 cells. The total amount of transfected DNA was kept constant by adding control vector pcDNA3-SRα/FLAG. The luciferase activity was analyzed at 48 h post transfection. The relative luciferase activity was presented as fold change relative to that of control transfection. Experiments were performed at least three times, and the error bar indicates ±1 s.d. of the mean. Statistical analyses were carried out using *t* test (*, p < 0.05; **, p < 0.01; ***, p < 0.001).

Supplementary Figure S5. DDX3 differentially regulates expression of DNMT3A-responsive genes. mRNA expressions of PTEN, RASSF1A, p53, RB1 and GAPDH in shLuc, shDDX3 #2 and shDDX3 #3 cells were detected by qRT-PCR. GAPDH was used as internal control. Fold change of each mRNA transcript in shDDX3 #2 and shDDX3 #3 cells was relative to that of shLuc cells. Results are derived from at least three independent experiments, and the error bar indicates ±1 s.d. of the mean. Statistical analyses were carried out using *t* test (*, $p < 0.05$; **, $p < 0.01$).

Supplementary Method

Assay of reporter plasmid activity

The luciferase reporter construct containing *MIR200B* promoter (-1574 to +120, relative to the putative transcription start site)[1] was kindly provided by Dr. Gregory J. Goodall (Centre for Cancer Biology, SA Pathology and University of South Australia, Australia). Reporter construct as well as different amounts of plasmid pcDNA3-SRα/FLAG or pcDNA3-SRα/FLAG-DDX3 were cotransfected into HepG2 cells (3×10^5 cells/well in a 6-well plate) using TransIT-LT1 transfection reagent according to the manufacturer's instruction (Mirus Bio LLC, Madison, WI, USA). At 48 h post transfection, the luciferase activity was assayed as described previously[2].

1. Bracken, C.P. *et al.* A double-negative feedback loop between ZEB1-SIP1 and the microRNA-200 family regulates epithelial-mesenchymal transition. *Cancer Res.* **68**, 7846-7854 (2008).
2. You, L.R., Chen, C.M. & Lee, Y.H. Hepatitis C virus core protein enhances NF-kappaB signal pathway triggering by lymphotoxin-beta receptor ligand and tumor necrosis factor alpha. *J. Virol.* **73**, 1672-1681 (1999).

Supplementary Tables

Supplementary Table S1. Clinicopathological information of HCC patients obtained from the TLCN

Parameter		No. of Patients (n = 39)
**Gender**		
	Male	24 (61.5%)
	Female	15 (38.5%)
**Hepatitis virus**		
	HBV	12 (30.8%)
	HCV	15 (38.4%)
	NBNC*	12 (30.8%)
**Tumor grade**		
	Grade 1	3 (7.7%)
	Grade 2	25 (64.1%)
	Grade 3	11 (28.2%)
**Tumor size (cm)**		
	≦5	24 (61.6%)
	>5	15 (38.4%)
**AFP (ng/ml)**		
	≦400	17 (43.6%)
	>400	22 (56.4%)
**Cirrhosis**		
	Absence	25 (64.1%)
	Presence	14 (35.9%)
**Vascular invasion**		
	Absence	17 (43.6%)
	Capsular vein	1 (2.6%)
	Portal vein	21 (53.8%)
**Pathological stage**		
	I	16 (41.0%)
	II	16 (41.0%)
	IIIA-IIIC	7 (18.0%)
**DDX3**		
	Non-reduced	9 (23.1%)
	Reduced	30 (76.9%)
**Age (mean ± SE)**		62.8 ± 10.9

*: non-HBV and non-HCV

Supplementary Table S2. qRT-PCR primers for mRNA expression

Gene	Primer sequence
DDX3	F: GAAGCTACTAGAGGTTTCTAC<br>R: TCTCAACATCACTGAAACTTTC
CD133	F: ACATGAAAAGACCTGGGGG<br>R: GATCTGGTGTCCCAGCATG
CD13	F: CATCAGCATTACCAACAAC<br>R: CATACTCGGTGGAGAATC
EpCAM	F: CTGGTGTTATTGCTGTTATTGT<br>R: CATTTGCTATTTCCCTTCTTCTAT
CD90	F: CATCTCCAGCATTCTCAG<br>R: TTACCTCCTTCTCCAACC
DNMT3A	F: TATTGATGAGCGCACAAGAGAGC<br>R: GGGTGTTCCAGGGTAACATTGAG
DNMT3B	F: GGCAAGTTCTCCGAGGTCTCTG<br>R: TGGTACATGGCTTTTCGATAGGA
DNMT1	F: TACCTGGACGACCCTGACCTC<br>R: CGTTGGCATCAAAGATGGACA
PTEN	F: TGGATTCGACTTAGACTTGACCT<br>R: TGGCGGTGTCATAATGTCTTTC
RASSF1	F: GGCGTCGTGCGCAAAGGCC<br>R: GAACCTTGATGAAGCCTGTG
p53	F: GCGCACAGAGGAAGAGAATC<br>R: CTCTCGGAACATCTCGAAGC
RB1	F: CATCGAATCATGGAATCCCT<br>R: GGA AGATTAAGAGGACAAGC
GAPDH	F: CACCCACTCCTCCACCTTT<br>R: TCCACCACCCTGTTGCTGTAG

Supplementary Table S3. qRT-PCR assay ID for miRNA expression

miRNA name	Assay ID
hsa-miR-200b	mature: 002251<br>primary: Hs03303027_pri
hsa-miR-200c	mature: 002300<br>primary: Hs03303157_pri
hsa-miR-122	mature: 002245<br>primary: Hs03303072_pri
hsa-miR-145	mature: 002278<br>primary: Hs03303169_pri
hsa-miR-10b	mature: 002218<br>primary: Hs03302879_pri
hsa-miR-519a	mature: 002415<br>primary: Hs03295478_pri
U6 snRNA	001973

Supplementary Table S4. qRT-PCR primers for ChIP assay and methylated DNA enrichment assay

miRNA name	Primer sequence
hsa-miR-200b	F:GAGCCCAGGGGACACACCT<br>R:CTCGCCTTACAAGGAGCAGT
hsa-miR-200c	F:AGGGGTGAGACTAGGCAGGT<br>R:CCACTGCCTTAACCCCTTC
hsa-miR-122	F:GCAGATAAGGAGGAGCTTCAGAC<br>R:AAGAGTCACCGGTCACAGGAGTGG
hsa-miR-145	F:CCGTAATTGGCTGAGCGTGG<br>R:GAGACAGGGTTTCACCGTGG
